# Assessing the Acceptability of Science Operations Concepts and the Level of Mission Enhancement of Capabilities for Human Mars Exploration Extravehicular Activity

**DOI:** 10.1089/ast.2018.1912

**Published:** 2019-03-06

**Authors:** K.H. Beaton, S.P. Chappell, A.F.J. Abercromby, M.J. Miller, S.E. Kobs Nawotniak, A.L. Brady, A.H. Stevens, S.J. Payler, S.S. Hughes, D.S.S. Lim

**Affiliations:** ^1^ KBRwyle, Houston, Texas, USA.; ^2^ NASA Johnson Space Center, Houston, Texas, USA.; ^3^ Jacobs Technology, Houston, Texas, USA.; ^4^ Department of Geosciences, Idaho State University, Pocatello, Idaho, USA.; ^5^ School of Geography and Earth Sciences, McMaster University, Hamilton, Ontario, Canada.; ^6^ European Astronaut Centre, European Space Agency, Cologne, Germany.; ^7^ BAER Institute, Moffett Field, California, USA.; ^8^ NASA Ames Research Center, Moffett Field, California, USA.

**Keywords:** Extravehicular activity, Planetary analogs, Operations concepts, Science operations, Human spaceflight, Communication latency and bandwidth

## Abstract

The Biologic Analog Science Associated with Lava Terrains (BASALT) research project is investigating tools, techniques, and strategies for conducting Mars scientific exploration extravehicular activity (EVA). This has been accomplished through three science-driven terrestrial field tests (BASALT-1, BASALT-2, and BASALT-3) during which the iterative development, testing, assessment, and refinement of concepts of operations (ConOps) and capabilities were conducted. ConOps are the instantiation of operational design elements that guide the organization and flow of personnel, communication, hardware, software, and data products to enable a mission concept. Capabilities include the hardware, software, data products, and protocols that comprise and enable the ConOps. This paper describes the *simulation quality* and *acceptability* of the Mars-forward ConOps evaluated during BASALT-2. It also presents the *level of mission enhancement* and *acceptability* of the associated Mars-forward capabilities. Together, these results inform science operations for human planetary exploration.

## 1. Introduction

### 1.1. The BASALT research project

The Biologic Analog Science Associated with Lava Terrains (BASALT) project is a science-driven research program designed to enable and enhance exploration and discovery during future human planetary missions. BASALT comprises *Science, Science Operations,* and *Technology* objectives. BASALT Science is focused on understanding habitability conditions of early and present-day Mars through the study of two Mars analog locations on Earth: the Kilauea Volcano 1969–1974 flows on the Big Island of Hawai‘i (early Mars analog) and the Eastern Snake River Plain in Idaho (present-day Mars analog) (Hughes *et al.,* 2019). The primary Science objective is to characterize and compare the physical and geochemical conditions of life in these environments and to learn how to seek, identify, and characterize life and life-related chemistry during these two epochs of martian history (Hughes *et al.,* 2019; Lim *et al.,* 2019).

BASALT Science Operations and Technology seeks to evaluate concepts of operations (ConOps) and capabilities for their potential to enhance scientific return during future exploration extravehicular activity (EVA) on planetary bodies. The ConOps and capabilities prioritized for investigation during BASALT are based on the composite results and lessons learned from previous exploration analog missions, including the Desert Research and Technology Studies (DRATS), NASA Extreme Environment Mission Operations (NEEMO) 18–21, and the Pavilion Lake Research Project (PLRP) (see Beaton *et al.,* 2019, for an overview of the significant accomplishments and forward-work recommendations from these prior analogs). BASALT Science Operations also incorporates current NASA architectural assumptions for future human planetary missions, including long-duration (*i.e.,* multiyear) flights, limited crew size (*e.g.,* 4–6), and space-to-ground (SG) communication delays and data transfer limitations.

Joint human-robotic exploration of Mars will enhance geologic sampling abilities through the strategic design and inclusion of ConOps and capabilities that best take advantage of humans' presence on the surface (NASA, 2015; Crusan *et al.,* 2017; ISECG, 2018; NAC, 2018). Hence, the primary goals of BASALT Science Operations are to inform requirements for human exploration EVA ConOps, provide recommendations for technology design and mission implementation and integration, and drive out future testing needs to ultimately enable safe, efficient, and effective scientific exploration (Beaton *et al.,* 2019).

BASALT Science, Science Operations, and Technology aims were addressed through three operational field tests during which Mars-relevant scientific exploration of terrestrial lava terrains was conducted under simulated Mars mission conditions. BASALT-1 took place in Idaho in June 2016, and BASALT-2 and BASALT-3 took place in Hawai‘i in November 2016 and November 2017, respectively. Details of BASALT-1 field operations and a subset of the science operations results are presented in the work of Beaton *et al.* (2017). Beaton *et al.* (2019) describes the BASALT-2 baseline ConOps and capabilities, overall mission architecture, and field test execution in detail; their article also presents some of the BASALT-2 Science Operations objective results, including data on EVA timeline and traverse execution and SG interactions between the “Mars-based” EVA crewmembers and “Earth-based” scientists. This paper is a companion to that of Beaton *et al.* (2019) and focuses on the Science Operations subjective results, including the *acceptability* of the ConOps, systems, and communication protocols investigated during BASALT-2 and the *level of mission enhancement* and *acceptability* of the associated capabilities with respect to future Mars EVA.

### 1.2. BASALT Science Operations

The BASALT Science Operations team is examining five strategic research questions that build upon the culmination and continuation of previous exploration analog studies and are aligned with larger strategic interests for future planetary EVA. NASA maintains an Integrated EVA Human Research Plan for coordination and collaboration among three of the primary participants in EVA research at the Johnson Space Center (JSC): the EVA Office, the Crew and Thermal Systems Division, and the Human Research Program (Abercromby *et al.,* 2015). Knowledge gaps identified in this plan call for a better understanding of the methods and tools required to operate across communication latency between Earth and Mars. The results gleaned from the BASALT project will contribute to future mission planning, management, and execution.

Previous NASA analog programs have focused on the evaluation of various ConOps and technologies relevant to planetary EVA operations (see Beaton *et al.,* 2019, for details). However, BASALT is the first NASA analog project to incorporate Mars-relevant field science into the testing and evaluation of planetary EVA operations. Furthermore, BASALT incorporates a rigorous approach to evaluate the success, or lack thereof, of critical components of the tested operations to meet scientific objectives (detailed in Section 2).

The BASALT Science Operations research questions are as follows:

- **Science Operations 1A**: Do the baseline Mars mission ConOps, systems, and communication protocols developed and tested during previous NASA analog tests work acceptably during real scientific field exploration? What improvements are desired, warranted, or required?
- **Science Operations 1B**: Do these ConOps, systems, and communication protocols remain acceptable as communication latency increases from 5- to 15-min one-way light time (OWLT)? What improvements are desired, warranted, or required?
- **Science Operations 2A**: Which capabilities enable and enhance Mars scientific exploration EVA?
- **Science Operations 2B**: Do these capabilities remain enabling and enhancing as communication latency increases from 5- to 15-min OWLT?
- **Science Operations 2C**: Do these capabilities remain enabling and enhancing as communication bandwidth allowances decrease?

*ConOps* are the instantiation of operational design elements that guide the organization and flow of personnel, communications, hardware, software, and data products to enable a mission concept. Operational design elements include the specific tasks that need to be accomplished and how they are organized into timelines and traverses; the roles, responsibilities, and distribution of personnel on Mars and on Earth; and flight rules that govern operations and safety. *Systems* refer to the hardware, software, and data products that support the ConOps. *Communication protocols* include the number and type of communication channels (*e.g.,* SG-1 and SG-2, described in Section 1.3), the mode of communication (*e.g.,* voice, text message, video, still imagery, instrument data), the temporal aspect of communication (*e.g.,* real-time vs. delayed), and the senders and receivers (*e.g.,* EV crew, IV crew, MSC scientists). *Capabilities* refer to the superset of operational elements, systems, and protocols that comprise and enable the ConOps.

The ConOps evaluated during BASALT focus on planetary EVA that integrates Earth-based scientific expertise *within an EVA* under Mars-relevant SG communication latencies and bandwidth constraints. While other ConOps (*e.g.,* those that focus on *between* or *inter*-EVA Earth-based inputs) are certainly viable for future human missions, *intra*-EVA SG interactions are advantageous when science objectives include exploring many regions of interest (the total number of which will necessarily be constrained by transport costs and EVA consumables) or when science objectives limit repeat visits to a particular location (*e.g.,* to reduce the potential for cross-contamination). Furthermore, the evaluation of ConOps that incorporate intra-EVA SG interactions directly addresses the presumption that latency and bandwidth constraints associated with missions beyond the Earth-Moon system preclude meaningful intra-EVA communication with Earth and hence require nearly complete crew autonomy (Pohlkamp *et al.,* 2015; Frank *et al.,* 2016). From an EVA perspective, this translates into an assumption that SG interactions within an EVA, especially in the form of tactical scientific guidance, become more challenging as SG communication latency increases and data transmission allowance decreases. BASALT seeks to evaluate the ability of a team of expert, remote scientists to effectively and efficiently enhance scientific return conducted by extravehicular and intravehicular crewmembers under Mars-relevant SG communication constraints.

### 1.3. BASALT-2 baseline architecture

The BASALT-2 field test consisted of 10 planned simulated EVAs: eight primary EVAs, two for each of the four communication study conditions described below, and two backup EVAs, in the event that poor weather or significant simulation anomalies occurred. Each EVA was approximately 4 h in duration, with predefined scientific observation and sampling objectives from the Mauna Ulu region of the Kilauea volcano (see Hughes *et al.,* 2019).

Extravehicular activities included two extravehicular (EV) crewmembers (EV1 and EV2) in the field conducting the science tasks and two intravehicular (IV) crewmembers (IV1 and IV2) inside a simulated habitat providing real-time support to the EV crew. EV1 and IV1 led the operational aspects of the EVA, including navigation and timeline management, while EV2 and IV2 led the science aspects of the EVA. The IV crew communicated across Mars-relevant time delays with an “Earth-based” Mission Support Center (MSC). The MSC consisted of expert scientists and engineers who received various data products from the field across the time delay and provided scientific and operational guidance to the crew. Notable MSC roles included a Flight Director who had ultimate authority over operational recommendations to the crew from the MSC, an EVA planner who relayed critical bingo times to the MSC (*i.e.,* countdowns until key pieces of information needed to be sent from the MSC to the crew to minimize the chances of crew idle [*i.e.,* nonproductive] time, described further in Section 1.4), a Science Team lead who coordinated the priorities and decisions from the geology and biology science subteams within the MSC, and a science communicator (SCICOM) who distilled and relayed science team recommendations to the crew. Table 1 of Beaton *et al.* (2019) defines the individual roles and responsibilities of the EV, IV, and MSC personnel.

**Table 1.** Key Mars-Forward Capabilities Incorporated and Evaluated during BASALT-2

*Category*	*Capability*	*Features and Functions*	*BASALT-2 Implementation*
**Pre-EVA: EVA Planning**	EVA traverse planning	Precursor imagery similar to current best-available for Mars and traverse planning software for designing EVA planned traverses	Google Earth imagery at 0.15 m/pix, multispectral imagery at 2.0 m/pix, and DEM at 10 m/pix imported into xGDS and SEXTANT to generate planned traverses (Marquez *et al.,*^2019^)
	EVA timeline planning	Precursor imagery similar to current best-available for Mars and timeline planning software to estimate EVA phase durations	Google Earth imagery at 0.15 m/pix, multispectral imagery at 2.0 m/pix, and DEM at 10 m/pix imported into xGDS, SEXTANT, and Playbook to generate planned EVA phase durations (Marquez *et al.,*^2019^)
	Planned traverse path optimization	Optimization of planned traverses by minimization of cost function based on, *e.g.,* terrain slope and projected crewmember metabolic cost	SEXTANT use of 0.03–0.22 m DEMs and a metabolic energy expenditure model to calculate optimal path (*i.e.,* no nontraversable slopes and minimal energy expenditure); optimal path displayed via Google Earth on EV wrist display and in xGDS for IV/MSC (Marquez *et al.,*^2019^)
**Intra-EVA: Voice and Text Communi-cation**	Voice comm.: EV crew ↔ IV crew	Real-time voice communication among EV and IV crewmembers	EV and IV wore monaural, noise-cancelling earbuds with boom mics; real-time voice comm. was transmitted across the BASALT comm. network (Miller *et al.,*^2019^)
	Voice comm.: IV crew ↔ MSC	Time-delayed voice communication between IV crew and MSC personnel	IV and CAPCOM/SCICOM wore monaural, noise-cancelling earbuds with boom mics; time-delayed voice comm. was transmitted across the BASALT comm. network (Miller *et al.,*^2019^)
	Voice transmission: EV/IV conversation → MSC	Time-delayed transmission of EV/IV crew conversations to MSC	Time-delayed transmission of EV/IV crew conversations to MSC were sent across the BASALT comm. network (Miller *et al.,*^2019^)
	Text comm.: IV crew ↔ MSC	Time-delayed text messaging communication between IV crew and MSC	Time-delayed text messages between IV crew and MSC were exchanged via Playbook Mission Log (Marquez *et al.,*^2019^)
**Intra-EVA: Video, Still Imagery, and Scientific Instrument Data from the Field**	Video from EV crew chest-mounted camera	Chest-level video footage transmitted from field to IV workstation (real-time) and MSC (time-delayed) for contextual and close-up applications	EV crew wore chest-mounted video cameras (1920 pix × 1080 pix resolution, 70.5° hFOV × 43.3° vFOV, 30 frames/s), which transmitted footage to IV workstation and MSC (bandwidth permitting) across the BASALT comm. network (Miller *et al.,*^2019^)
	Mobile SA video with position and orientation tracking	Mobile SA video footage of EV crew in their environment transmitted to IV workstation (real-time) and MSC (time-delayed); position and orientation tracking of SA camera; camera at a height of ∼3–4 m, with pan-tilt-zoom capabilities, and able to be controlled remotely by IV	FST manually followed EV crew with SA video camera (1920 pix × 1080 pix resolution, 70.5° hFOV × 43.3° vFOV, 30 frames/s) mounted on ruggedized, lightweight tripod at height of ∼1–2 m. Footage was sent to IV workstation and MSC (bandwidth-permitting) across the BASALT comm. network (Miller *et al.,*^2019^)
	High-resolution still imagery	High-resolution imagery transmitted from field to IV workstation (real-time) and MSC (time-delayed)	EV crew used handheld point-and-shoot cameras to capture still imagery at 8 MP (under high-bandwidth conditions) and 3 MP (under low-bandwidth conditions); images were sent to IV workstation (real-time) and MSC (time-delayed) across the BASALT comm. network and automatically imported into xGDS (Brady *et al.,*^2019^; Marquez *et al.,*^2019^; Miller *et al.,*^2019^; Stevens *et al.,*^2019^)
	Handheld scientific instrument data	Capture thermal imagery, mineral identification, and elemental composition of specific targets in the field, display data to EV crew, and transmit data to IV crew (real-time)	EV crew used handheld FLIR E60 camera (temp range: −20°C to 650°C, sensitivity: 0.05°C, accuracy: ±2°C, thermal image resolution: 320 pix × 240 pix, RGB image resolution: 2048 pix × 1536 pix), ASD Terraspec Halo vis-NIR spectrometer (350–2500 nm), and Bunker Tracer IV-SD XRF spectrometer (reflectance spectra from 350–2500 nm) (Sehlke *et al.,*^2019^); EV crew captured still images of FLIR and ASD data screens, which were sent to IV workstations (real-time) and MSC (time-delayed) across the BASALT comm. network and automatically imported into xGDS; raw XRF data was transmitted across the BASALT comm. network to the IV workstation and MSC (Marquez *et al.,*^2019^; Miller *et al.,*^2019^)
**Intra-EVA: EV Crew Support Tools**	Navigation aids	Provide EV crew with current position, planned path, actual path, waypoints of interest, and mapped notes	Graphical display (see below) used to show EV crew current position, planned traverse path, actual traverse path, waypoints of interest, and mapped notes from xGDS (Marquez *et al.,*^2019^)
	Graphical display	Graphical display that presents navigation aids, text messages, annotated images, and FOV of EV chest-mounted video camera transmission	iPhone 6 Plus attached to forearm with armband case that displayed Google Earth containing planned and actual traverses, waypoints of interest, and mapped notes, Playbook Mission Log, and TerraDek for viewing of video camera transmission (Miller *et al.,*^2019^)
	Feature pointer	Physical pointing stick to designate features of interest in the field that provides sense of scale and orientation relative to surrounding terrain	Handheld 1 m long, 1 in. diameter white PVC pipe used by EV crew to point at features of interest in video feeds and still imagery; one end of stick included alternating 2 cm black and white stripes for cm-scale resolution scale bar in imagery; stick used in combination with handheld compass to designate cardinal direction in imagery
	Feature marker	Physical marker with unique label to unambiguously mark and identify a terrain feature of interest that incorporates scale bar, color bar, and orientation designator	3D printed yellow cards (10 cm × 15 cm × 0.3 cm) with candidate sample ID (*e.g.,* AB designating the station [A] and candidate within the station [B]), 2 cm black hash marks for cm-scale resolution scale bar, 18-component grayscale and color bar, and arrow that can rotate to align with cardinal direction (Stevens *et al.,*^2019^)
**Intra-EVA: IV and MSC Support Tools**	Geospatially linked electronic field notes	Electronic field notes captured by IV and MSC that are visible to all and linked geospatially to EV crew positions at the traverse map; time that the note is recorded	xGDS mapped field notes (Marquez *et al.,*^2019^)
	Dynamic leaderboard	Candidate presampling and sampling ranking system that incorporates candidate IDs, measure of strength of each ranking, rationale associated with each ranking, and photos of each candidate	Google Sheet with one row associated with each candidate sample, rows organized by highest to lowest priority, and columns for candidate marker ID, candidate descriptions, candidate photos, and priority ranking rationale (see Fig. 1 in Stevens *et al.,*^2019^)
	Spatial and temporal synchronization of field data	Spatial and temporal synchronization of EV crew GPS positions, still imagery, instrument data, and field notes	Spatial and temporal synchronization through xGDS and Playbook (Marquez *et al.,*^2019^)
	Image annotation	Image annotation (*e.g.,* circling features of interest, adding arrows, adding text) by MSC with ability to transmit annotations to EV and IV crew	Still imagery exported out of xGDS and into Microsoft PowerPoint for image annotation; annotated images transmitted as jpg's via Playbook Mission Log (Stevens *et al.,*^2019^)
	Tactical EVA timeline management	Dynamic EVA timeline for use by IV crew and MSC that displays sequence of planned EVA phases and planned EVA phase durations, records actual phase durations, projects future phase start times based on actual phase durations, displays countdown timers for key deadlines, and provides space for taking notes associated with each EVA phase	Microsoft Excel spreadsheet containing columns for EVA phase durations, planned phase start times, projected phase start times (based on actuals), phase descriptions, notes for each EV crewmember, and running clocks that display current time, PET, phase time remaining, overall EVA time remaining. MSC version also contained countdown timers for when critical pieces of information needed to be sent to IV crew to minimize chances of crew idle time. Clocks and timers were automatically color-coded green and red to display ahead or behind the planned timeline, respectively.
	+/− ∼1 m position tracking	Track and display position of EV crew and locations of interest to EV, IV, and MSC to +/− m-level resolution	GPS position tracking of EV crew and terrain features of interest via EV backpack-mounted GPS units transmitting position to xGDS for display to IV/MSC and to Google Earth for display to EV via graphical wrist display (Marquez *et al.,*^2019^; Miller *et al.,*^2019^)
	EVA traverse replanning	Augment precursor data with incoming data from the field to replan EVA traverses	Precursor data plus EV crew verbal descriptions, still imagery, video footage, and scientific instrument data from the field to replan EVA traverses
	EVA timeline replanning	Augment precursor data with incoming data from the field and actual EVA phase durations to replan EVA timelines	Precursor data plus projections made by the EV/IV crew and MSC (based on EV crew verbal descriptions, still imagery, video footage, and scientific instrument data from the field plus actual EVA timeline durations as measured by the tactical EVA timeline management tool) to replan EVA timelines
**Post-EVA: Archiving**	EVA data storage	Organized storage of all voice, text, video, still imagery, instrument data, and field notes	xGDS stored precursor planning data, planned and actual traverses, still imagery, and field notes; Playbook stored planned and actual EVA timelines and time-stamped Mission Log entries; voice and video data stored on separate external server (Marquez *et al.,*^2019^; Miller *et al.,*^2019^)

3D: three dimensional, CAPCOM: capsule communicator, DEM: digital elevation model, EV: extravehicular, EVA: extravehicular activity, FLIR: forward-looking infrared, FOV: field of view, GPS: global positioning system, hFOV: horizontal field of view, ID: identifier, IV: intravehicular, MP: megapixel, MSC: mission support center, PET: phased elapsed time (*i.e.,* time since start of EVA), pix: pixel, PVC: polyvinyl chloride, RGB: red, green, blue, SA: situational awareness, SCICOM: science communicator, vFOV: vertical field of view, vis-NIR: visible to near infrared, xGDS: Exploration Ground Data System, XRF: X-ray fluorescence.

The Kilauea Military Camp, located inside the Hawai‘i Volcanoes National Park, supported the BASALT team throughout the field test. They provided a large conference room to house the MSC during the EVAs and a small, isolated side room that served as the simulated Mars habitat for the IV crew. Figure 2 of Lim *et al.* (2019) depicts the organization and distribution of EV, IV, and MSC personnel in Hawai‘i and in relation to an actual Mars mission.

To increase the number of participants for consensus discussions (described in Section 2) and to minimize field test fatigue, three EVA teams were established, including three pairs of EV and IV crewmembers, three Science Team Leads, three SCICOMs, and two sets of the other MSC operators and scientists. The personnel assigned to these teams came from the BASALT team of investigators, and all had extensive experience in science and/or operations through other fieldwork and previous spaceflight analog programs. Teams were counterbalanced across four different communication study conditions.

BASALT-2 EVAs were conducted under one of four communication study conditions: (1) 5-min OWLT latency + “high” bandwidth (5.0 Mb/s uplink and 10.0 Mb/s downlink); (2) 5-min OWLT latency + “low” bandwidth (0.512 Mb/s uplink and 1.54 Mb/s downlink); (3) 15-min OWLT latency + “high” bandwidth; and (4) 15-min OWLT latency + “low” bandwidth. The 5- and 15-min latencies fall within the 4–22 min OWLT delays experienced between Mars and Earth. The low-bandwidth condition represents a conservative and lower-cost flight data rate, while the high-bandwidth condition represents an upgraded capability that would require additional infrastructure and technology development (Rush *et al.,* 2012; NASA, 2015; Seibert *et al.,* 2019). Intra- and post-EVA network analytics were completed to ensure bandwidth traffic stayed within the low and high bandwidth limits (Miller *et al.,* 2019).

The following baseline capabilities facilitated communication and data transmission among the EV crew, IV crew, and the MSC, and enabled the scientific exploration and sampling to be completed. A summary of these capabilities, their associated features and functions, and how they were implemented in BASALT-2 is provided in Table 1.

Baseline capabilities included real-time (between EV and IV crew) and delayed (between EVA crew and the MSC) transmission of voice data, text messages, video footage from EV crew chest-mounted cameras and from a mobile situational awareness (SA) camera, high-resolution still imagery from EV crew handheld point-and-shoot cameras, scientific data from handheld field instruments, and GPS position data of the EV crew and mobile SA camera. During the EVAs, the EV crewmembers wore custom extravehicular informatics backpacks (EVIB) that contained the hardware needed to enable these baseline capabilities (see Miller *et al.,* 2019). They also wore graphical wrist displays to view their GPS position tracks, text messages and annotated images from IV crewmembers and the MSC, and video feeds streaming from their chest-mounted cameras. The EV crew had a series of science tools, including candidate sample location markers, feature pointers, handheld scientific instruments, and sterile sampling tools (Stevens *et al.,* 2019). The BASALT project originally planned for the inclusion of a Mobile Instrument Platform (MIP), consisting of a simulated rover mast-mounted SA camera, high-resolution panoramic camera, and mobile automated light detection and ranging (LiDAR) instrument that could be remotely operated by the IV crew. However, due to time and resource limitations associated with BASALT-2, only the mobile SA camera component of the MIP was incorporated into this field test.

The IV crew and the MSC were supported by Minerva, an integrated science and operations support tool comprised of the Exploration Ground Data System (xGDS) software package (Deans *et al.,* 2017), Playbook timeline management tool (Marquez *et al.,* 2013), and SEXTANT traverse optimization tool (Marquez *et al.,* 2019). Minerva enables the creation, modification, and display of EVA traverses and timelines and provides a sophisticated science operations management platform and repository for scientific data, imagery, and field notes (Marquez *et al.,* 2019).

Two primary SG communication channels facilitated data transfer between the EVA crew and the MSC: SG-1 and SG-2 (Abercromby *et al.,* 2013a; Chappell *et al.,* 2013a). EV and IV crew conversations and data from the field (including video footage, still imagery, and science instrument data) were transmitted to the MSC across latency on SG-1. The MSC and IV crew conversed (primarily via text messages but occasionally via voice) across SG-2. The EV crew did not listen to SG-2, and the MSC did not converse with the crew across SG-1.

Because an automated bandwidth management strategy was not in place for BASALT-2, low-bandwidth EVAs limited the transmission of video footage and high-resolution still imagery from the field to the MSC. Specifically, EV crew chest camera and mobile SA camera video footage was not transmitted to the MSC (although these feeds were sent to the IV crewmembers), and lower-resolution still imagery (3 MP images, as opposed to 8 MP images under high-bandwidth conditions) was captured and transmitted by the EV crew.

### 1.4. BASALT-2 EVA execution

BASALT EVAs incorporate several key assumptions relevant to human Mars missions. First, it is assumed that for exploration destinations such as Mars, robotic precursor missions will have collected relevant precursor data to plan science exploration traverses to be conducted by human crews. This information could be collected by overhead satellites, surface rovers, surface unmanned aerial vehicles, or previous crew visits (ISECG, 2018).

BASALT-2 precursor data included Google Earth imagery at a resolution of 0.15 m/pix, multispectral imagery at ∼2 m/pix, and digital elevation models (DEMs) at 10 m/pix; this data is similar to that provided by current Mars orbital assets, including the Mars Reconnaissance Orbiter (MRO) High Resolution Imaging Science Experiment (HiRISE), Mars Express High Resolution Stereo Camera (HRSC), and Mars Global Surveyor (MGS) Mars Orbiter Laser Altimeter (MOLA) (Jaumann *et al.,* 2007; McEwen *et al.,* 2007; Brady *et al.,* 2019). BASALT scientists used the precursor visible imagery to identify terrain types, spatial relationships between volcanic features, and overall geologic context. The multispectral false-color product was used to identify potential sampling target sites having various states of oxidation and alteration: unaltered basalt, syn-emplacement altered basalt, and active and relict fumaroles. The DEMs were used to gauge slopes and identify safe traverse paths for the EVAs. From this, the scientists identified candidate locations of scientific interest (referred to as EVA *stations,* each of which was approximately 10 m in diameter) and designed baseline EVA traverses (the routes covered by the EV crewmembers) in advance of the field test. A summary of the BASALT-2 baseline EVA traverses is provided in Fig. 1 of Beaton *et al.* (2019).

**FIG. 1.**
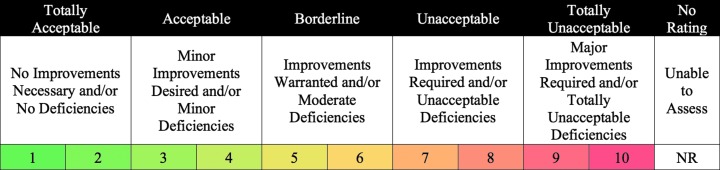
EAMD acceptability rating scale.

The second assumption is that upon execution of an EVA by the EV crew, additional information (*i.e.,* beyond that provided by precursor data) will be obtained for each location of interest that may result in modifications to the original traverse plans, science tasks, and/or science priorities. The purpose of conducting exploration EVAs by human crews is to obtain as much scientific information as possible to meet mission science objectives.

The third assumption is that a higher level of scientific expertise and analytical capabilities exists on Earth than with the crew. Although future Mars crewmembers will be highly trained, they may not be experts in all relevant scientific disciplines given the vast breadth and depth of science objectives expected for future human missions (Beaty *et al.,* 2015; Niles *et al.,* 2017). Crewmembers will, however, be trained to be effective observationalists; they will learn how to communicate what they find in the field back to Earth through whatever capabilities are at their disposal.

The fourth assumption is that EVA timelines can be strategically designed to allow for intra-EVA MSC input, even across substantial SG communication latencies and bandwidth constraints, without incurring crew idle (*i.e.,* nonproductive) time. BASALT-2 EVAs were designed to facilitate MSC expertise within an EVA under the 5- and 15-min SG communication delays and high- and low-bandwidth conditions. This was accomplished by knowing which tasks could be completed independent of MSC input and which tasks were either dependent on or could substantially benefit from MSC expertise.

For tasks benefiting from MSC input, dependent task groups were created and distributed throughout the timeline. With sufficient understanding of EVA task durations and dependencies (gleaned from prior fieldwork conducted by MSC scientists and operational readiness tests completed prior to each BASALT field test), communication latencies, and ground assimilation time (GAT, the amount of time that Earth-based scientists and operators have to make decisions affecting crewmembers' subsequent actions without the crew incurring idle time) and appropriate roles and responsibilities defined for the MSC, EVA timelines were created that allowed for MSC input on most tasks while avoiding crew idle time. Importantly, baseline timelines also maintained a flexible buffer to facilitate exploration within the initial survey and candidate sample search phases of the EVA so that crewmembers (and the MSC) could react to what was found in the field (Hodges and Schmitt, 2011).

During the EVAs, EV crewmembers explored the areas outlined by the baseline traverses. EVA timelines incorporated five phases: station approach, station contextual survey, station candidate sample location search, presampling, and sampling. A representative EVA timeline that includes the sequence and duration of EVA phases is provided in Table 2 of Beaton *et al.* (2019). Detailed tasks within each EVA phase included the EV crew providing verbal descriptions, video footage, high-resolution still imagery, and data from handheld scientific instruments; the final EVA phase included extracting samples of basalt that met that day's scientific objectives (see Sections 2.2 and 2.3 of Beaton *et al.,* 2019).

**Table 2.** High-Level Summary of Operational Status of Data Products Transmitted Between Space and Ground

EV: extravehicular, EVA: extravehicular activity, GPS: global positioning system, H: high, IV: intravehicular, L: low, MSC: mission support center, OWLT: one-way light time, pXRF: portable X-ray fluorescence, SA: situational awareness. Green = capability functioned reliably and consistently throughout the EVA; yellow = some instabilities occurred throughout the EVA; red = the capability did not function reliably or consistently during the EVA; gray = the capability was not exercised during the EVA.

The IV crewmembers were the critical liaison between the EV crew in the field and the MSC on Earth. They tracked the progress of the EV crew against the planned timeline and used their understanding of that day's science objectives to engage in an effective dialog with the MSC (across latency) regarding the candidate samples identified by the EV crew. The MSC was comprised of geology and biology subteams. The MSC Science Lead worked with the SCICOM to distill and relay science team priorities and recommendations to the crew; these messages were typically sent slightly “ahead of schedule” in case the crew worked faster than the planned timeline and to mitigate against potential communication dropout.

BASALT-2 EVAs were further supported by out-of-simulation (x-sim) support personnel. Because BASALT is not evaluating flight hardware or flight sampling techniques, an x-sim field support team assisted the EV crew with scientific instrument use (including carrying and handing off instruments at the appropriate times and troubleshooting malfunctions) and sterile sample collection. Two x-sim members of the BASALT communications and backpack team served as communication relays to provide communication coverage in the field and EVIB troubleshooting support. In the MSC, the simulation coordinator initiated the start and end of the EVAs, as well as any simulation pauses (*e.g.,* due to troubleshooting) within the EVAs. Additional communications infrastructure support, Minerva technical support, a science operations stenographer (responsible for manually recording detailed EVA task timing data), and a science stenographer (responsible for manually recording detailed EV crew comments) were available within the MSC.

Flight rules were established to govern aspects of BASALT-2 field testing, including in-simulation (in-sim) and x-sim operations. These flight rules provided the operating guidelines with respect to safety, mission management and authority, EVA management and authority, and troubleshooting. A mission management team that included the leads of each BASALT subteam (science, science operations, communications, EVIB, Minerva, and field support) was established to address concerns and necessary amendments to field operations during the field test and to ensure adherence to the flight rules throughout the field test. These flight rules are displayed in Table 3 of Beaton *et al.* (2019).

**Table 3.** Acceptability of the ConOps

EV: extravehicular, EVA: extravehicular activity, GAT: ground assimilation time, IV: intravehicular, MSC: mission support center, Ops: operations, Sci: science.

## 2. Methods

This paper describes the subjective assessments of *acceptability, capability assessment,* and *simulation quality* (Abercromby *et al.,* 2013b) of the ConOps and capabilities evaluated during BASALT-2. These metrics have been developed, refined, and vetted by the Exploration Analog and Mission Development (EAMD) team at NASA JSC. They have been successfully applied to the evaluation of habitability, human factors, and human performance aspects of candidate spaceflight vehicles (Litaker *et al.,* 2012, 2013; Litaker Jr. *et al.,* 2013; Gernhardt *et al.,* 2017) and to different operations concepts for future human exploration-class missions (Abercromby *et al.,* 2010, 2012, 2013b; Chappell *et al.,* 2011, 2013b, 2016) to derive actionable results and recommendations for future iterations and tests.

During BASALT-2, these assessments were collected real-time during brief (∼1 min) simulation pauses at the end of each EVA phase and during post-EVA consensus discussions, which occurred after the final EVA associated with each communication study condition and during final debrief sessions following the field test. Real-time ratings were collected individually by all EVA crewmembers and MSC personnel and served as memory aids for the consensus discussions. Consensus discussions were attended by all in-sim personnel, and separate consensus ratings were collected from the EVA crew and from the MSC to discriminate differences in perspective. The actionable results, recommendations, and forward work documented in Sections 3 and 4 stem from the data gathered during the consensus discussions.

### 2.1. Acceptability

*Acceptability* reflects the extent to which an operations concept or capability is considered an acceptable approach to conducting planetary exploration EVA and the extent to which improvements, if any, are *desired, warranted,* or *required* (Abercromby *et al.,* 2010). *Operational acceptability* is defined as the ability to effectively, efficiently, and reliably conduct operations with accurate exchange of all pertinent information and without excessive workload or (in-sim) avoidable inefficiencies or delay. *Scientific acceptability* is the ability to effectively and reliably complete and record scientific observations, measurements, and/or sampling with sufficient quantity, distribution, resolution, accuracy, and/or integrity to test the scientific hypothesis/hypotheses. Note that efficiency, or lack thereof, is covered under operational acceptability.

During BASALT-2, in-sim personnel were asked to provide their ratings and associated comments regarding how acceptable they believed an operations concept, protocol, or capability under evaluation would be for a real Mars human exploration mission. This required participants to extrapolate their experiences from the simulation (*i.e.,* from the BASALT EVAs) and to provide their best assessment of the extent to which the condition or element being evaluated would be acceptable during an actual spaceflight mission. This extrapolation was critical so that results would be as Mars-forward as possible (as opposed to BASALT-centric). Importantly, all questions regarding acceptability focused on evaluating the *features and functions* most critical for future spaceflight missions, not on evaluating specific implementations employed during BASALT-2 (see Sections 1.2 and 1.3 and Table 1 for the key features and functions associated with the ConOps, protocols, and capabilities that were assessed). If anything was found to be unacceptable, participants were asked to describe the specific improvements needed so that the element under evaluation could be deemed acceptable.

The acceptability rating scale was modified for the BASALT project from that used in previous analog tests to explicitly capture deficiencies in the ConOps and, where possible, identify potential improvements to address these deficiencies. In some cases, deficiencies were aspects that could be directly observed (*e.g.,* the inefficient use of EV crew time to read aloud results from a scientific instrument data screen to the IV crew and the MSC), and a corresponding improvement could be identified (*e.g.,* improve efficiency by directly transmitting the raw instrument data from the field to the IV workstation and the MSC). In other cases, deficiencies could be noted, but no improvement could be proposed (*e.g.,* loss of crew time when the MSC recommended a change in priorities while operating under long communication latencies—since communication latency cannot be controlled, no direct improvement can be identified). Finally, improvements in one aspect of a ConOps (*e.g.,* incorporating a presampling survey that is separate from and in advance of the sampling phase to allow for tactical, intra-EVA input by the MSC) could result in deficiencies in other aspects of the ConOps (*e.g.,* increased transportation cost required to move back and forth between different locations to enable presampling surveys).

The acceptability rating scale (displayed in Fig. 1) consists of five distinct categories: totally acceptable with no improvements necessary and/or no deficiencies, acceptable with minor improvements desired and/or minor deficiencies, borderline with improvements warranted and/or moderate deficiencies, unacceptable with improvements required and/or unacceptable deficiencies, and totally unacceptable with major improvements required and/or totally unacceptable deficiencies. Any rating of 4 or lower is considered acceptable. Any rating of 3 or higher requires comments as to what improvements are *desired, warranted,* or *required* so that the ConOps, protocol, or capability under evaluation would be deemed acceptable for future Mars EVA, and/or what deficiencies could be identified. A “no rating” is given either when the evaluator does not believe that they have sufficient experience with the concept being evaluated to provide a judicious rating or when the simulation quality associated with that concept or capability is deemed insufficient (see Section 2.4). Operational acceptability data was collected from EV1, IV1, and MSC operators; scientific acceptability data was collected from EV2, IV2, and MSC scientists.

### 2.2. Capability assessment

*Capability assessment* (CA) reflects the extent to which a capability (or potential capability) could be useful during a human exploration mission (Abercromby *et al.,* 2010). A primary objective of the BASALT project is to identify which capabilities are required for exploration EVA, which capabilities might enhance the EVA but are not essential, and which capabilities provide marginal or no meaningful enhancement and can therefore be excluded, resulting in cost savings without impact to mission success. Hence, we used the CA rating scale to evaluate the extent that candidate capabilities are expected to enable and enhance future human exploration missions. As with the acceptability evaluations, all questions regarding capability assessment focused on evaluating the *features and functions* most critical for future spaceflight missions, not on evaluating specific implementations employed during BASALT-2 (*e.g.,* we evaluated the level of mission enhancement for a Mars-forward capability that provides local positioning information at approximately 1 m resolution, as opposed to evaluating the details associated with an Earth-based GPS, which was employed during BASALT-2).

**FIG. 2.**
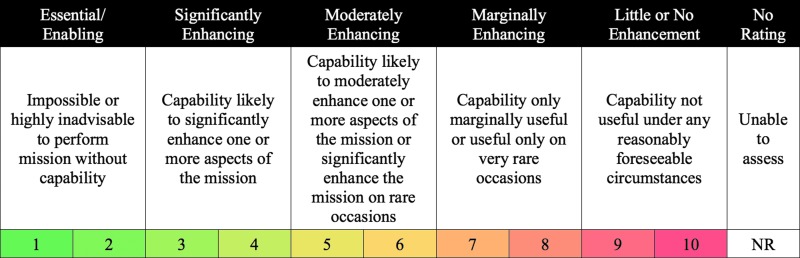
EAMD capability assessment rating scale.

The CA rating scale (shown in Fig. 2) consists of five categories: essential/enabling—impossible or highly inadvisable to perform a mission without the capability; significantly enhancing—capability is likely to significantly enhance one or more aspects of the mission; moderately enhancing—capability is likely to enhance one or more aspects of the mission or significantly enhance the mission on rare occasions; marginally enhancing—capability is only marginally useful or useful only on very rare occasions; and little to no enhancement—capability is not useful under any reasonably foreseeable circumstances. A “no rating” is given either when the evaluator does not believe that they have sufficient experience with the capability under evaluation to provide a judicious rating or when the simulation quality associated with that capability is insufficient (see Section 2.4).

Note that it is possible for a given capability to be rated totally acceptable on the acceptability rating scale but provide little or no mission enhancement from a capability assessment perspective, or vice versa. Hence, when rating a specific capability, both scales are used together to first evaluate the level of mission enhancement and then to identify desired, warranted, or required improvements that might make it acceptable for a future spaceflight mission.

For BASALT-2, CA ratings were collected from both a scientific and operational perspective by science crewmembers (EV2 and IV2) and MSC scientists and by operational crewmembers (EV1 and IV1) and MSC operators, respectively. Some capabilities were enhancing to both the science and the operations, while other capabilities were more enhancing to either the science or the operations.

### 2.3. Practical significance for acceptability and capability assessment ratings

The acceptability and CA ratings are based on 10-point Likert scales (Likert, 1932) divided into five distinct categories with two ratings within each category to discriminate preferences. These rating scales were developed to provide actionable recommendations under test conditions where sample sizes are typically not large enough to conduct traditional inferential statistics. Hence, we prospectively define *practically significantly different* to be a *categorical* difference on the scales (*e.g.,* the difference between a 4 and 5 on either the acceptability or CA scale) (Abercromby *et al.,* 2010). Intra-categorical differences discriminate smaller (non-practically significant) preferences (*e.g.,* the difference between a 3 and 4 on either the acceptability or CA scale). Importantly, comments are collected to capture the reasoning behind each numerical rating.

### 2.4. Simulation quality

*Simulation quality* reflects the extent to which a test enables meaningful evaluation of the study condition being evaluated (Chappell *et al.,* 2013b). Factors that might affect simulation quality include unplanned communication dropouts, low-fidelity hardware, or other factors that make the simulation unrealistic with respect to the study objective under consideration.

**FIG. 3.**
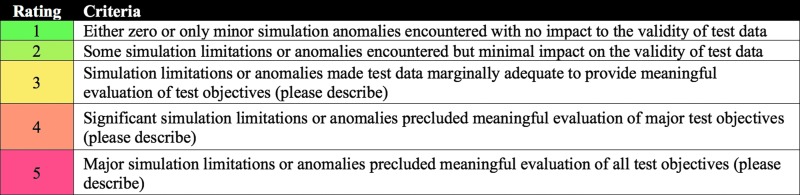
EAMD simulation quality rating scale.

The EAMD simulation quality rating scale is provided in Fig. 3. Any rating of 3 or higher requires a description of the simulation limitations. A simulation quality rating of 1 means that the test condition or objective under evaluation (*e.g.,* a particular ConOps or capability) is highly representative of an actual Mars mission condition or objective. A simulation quality rating of 3 means that some components may not be operating in a manner that is completely representative of a Mars mission, but there is sufficient fidelity for a meaningful evaluation to be reliably conducted. Simulation quality ratings of 4 or 5 mean that substantial simulation limitations exist, which preclude meaningful evaluation of those test objectives.

All acceptability and capability assessment questions are first preceded by a measure of simulation quality. If the simulation quality is insufficient to enable meaningful evaluation of the test objectives, no acceptability or CA data are collected. Note that the BASALT project is not evaluating spacesuits, flight hardware, or operations in reduced-gravity environments. Hence, these aspects were excluded from simulation quality evaluations; questions were instead focused on the features and functions of the ConOps, protocols, and capabilities that could enable and enhance a mission.

### 2.5. Comments, assumptions, and recommendations

A key feature of the EAMD assessment ratings process is the fact that assumptions, comments, and recommendations are documented for each numerical rating provided. While the numerical ratings are important and often the subject of considerable discussion and debate, their most important function is in providing structure and consistency to the process through which the different study conditions, ConOps, protocols, and capabilities are critiqued. In the case of acceptability ratings, specific limitations or improvements are required to be identified when any rating of 3 or higher is given; these documented observations and associated recommendations are the actionable results. Similarly, the methodology for rating simulation quality requires that any relevant limitations of the simulation are identified, which enables improvement of the simulation for future testing or else reprioritizes subsequent test conditions and assessments to evaluate only those conditions that can be adequately simulated.

## 3. Results

### 3.1. Simulation quality

Simulation quality ratings were collected in real-time at the end of each EVA phase by the EVA crewmembers and the MSC. These ratings were typically a 2 or a 3. During the consensus discussions following each communication study condition, it was determined that the overall simulation quality for each study condition was rated a 3, meaning that some simulation limitations or anomalies made the test data marginally adequate to provide meaningful evaluation of the baseline ConOps, systems, and communication protocols. There are two primary factors that led to overall simulation quality ratings of 3.

First, not all capabilities were functioning properly at all times throughout all EVAs. Table 2 provides a high-level summary of the operational status of the voice, text messaging, video, still imagery, GPS tracking, field note, and pXRF instrument data transmission capabilities for each EVA. Green means that the capability functioned reliably and consistently throughout the EVA, yellow means that some instabilities occurred throughout the EVA, red means that the capability did not function reliably during the EVA, and gray means that the capability was not used during the EVA (*e.g.,* video transmission to the MSC during low-bandwidth conditions). Note that numerous capabilities were red and yellow during EVA 1. For this reason, the overall simulation quality associated with EVA 1 was rated a 4, meaning that significant simulation limitations precluded meaningful evaluation of the test objectives. Hence, the communication study condition associated with EVA 1 was repeated on one of the backup EVA days.

Reliable data transmission between the field, IV workstation, and the MSC was occasionally challenging. Some areas of Mauna Ulu were more difficult to provide and maintain consistent communication network coverage because of the varied terrain that sometimes precluded line-of-sight with the various communication relay nodes (see Miller *et al.,* 2019). Additionally, occasional hardware failures, software glitches, or equipment overheating occurred in the field. In particular, the EV crew point-and-shoot cameras did not reliably connect to the EVIB to transmit still imagery back to the IV workstation and the MSC in a timely manner.

The second reason that overall simulation quality for the different study conditions was consistently rated a 3 was that some learning effects for the EVA crew and MSC personnel persisted throughout the field test. Three EVA teams were rotated throughout the analog mission to increase the number of individuals participating in consensus ratings discussions and to minimize fatigue caused by back-to-back EVA days, especially for the EV crewmembers. However, due to schedule and resource limitations, the training time available to all EVA and MSC personnel before the first EVA was limited to a week-long operational readiness test several months in advance of the field deployment (which took place in a simulated rock yard at the NASA Ames Research Center) and to three practice field days upon arrival in Hawai‘i; hence some individuals were still refining the manner in which they conducted their tasks during the actual EVA test days.

### 3.2. Acceptability and capability assessment

The BASALT team evaluated the operational and scientific acceptability of the ConOps, communication protocols, and capabilities with respect to future Mars exploration missions and identified *desired, warranted,* and *required* improvements, as well as significant deficiencies. Some improvements came in the form of recommending new capabilities, which were then evaluated with the CA rating scale; these results are presented in Section 3.3. The BASALT team also evaluated the level of mission enhancement of the baselined capabilities, outlined in Section 1.3, using the CA rating scale.

As described in Section 2, the acceptability and CA evaluations were collected separately by the EV/IV crew and by the MSC for each of the four communication study conditions. In some cases, there was no difference in the ratings or comments between the EVA crew and the MSC. Similarly, there were instances in which no difference was found in the ratings or comments across the different study conditions. Where differences were found, they are specified below. The results presented here are a combination of the results gathered during consensus discussions at the end of each study condition, consensus discussions at the end of the field test, and consensus discussions conducted one year after the field test to close out the data analysis.

#### 3.2.1. Acceptability of the ConOps

The ConOps under evaluation included the following operational design elements: *EVA tasks and their organization into timelines and traverses;* the *roles, responsibilities, and distribution of personnel on Mars and on Earth;* and *flight rules, specifically with respect to the advisory role of the MSC in EVA operations*. The scientific and operational acceptability and associated recommended improvements of each of these elements are presented in Table 3.

For *EVA tasks and their organization into EVA timelines and traverses,* the acceptability question of interest was “For future Mars missions with similar science objectives, how acceptable would it be to have similar EVA tasks organized into the EVA timelines and traverses employed during BASALT (*i.e.,* the BASALT EVA phases, timeline design to facilitate science team assimilation and input to the EV and IV crew, and traverse plan with multiple stations per EVA)?” In general, it was rated scientifically and operationally *borderline acceptable* to have timelines organized into the five BASALT EVA phases (approach, contextual survey, candidate search, presampling, and sampling). This structure, coupled with the available capabilities, enabled the MSC to assimilate and interpret incoming data from the field in time to provide meaningful recommendations to the crew.

However, it was found that the baseline stations did not always incorporate sufficient areas to explore to meet that day's science objectives; this was especially true for EVAs targeting relict fumaroles, whose properties were more difficult to identify with high confidence based on the precursor data available for planning. So, in some instances, the type and resolution of precursor data for BASALT-2 traverse planning were insufficient to provide the details needed to instill high confidence that the baselined 10-m diameter stations would ultimately house candidate samples that could meet the science objectives.

Figure 2 in Beaton *et al.* (2019) shows the planned versus actual timelines for each of the eight EVAs associated with simulation quality ratings of 3. The largest deviations in planned versus actual timelines (and also in planned versus actual traverses) occurred when the EV crew did not find what they expected upon arrival at a particular station and had to expand the station boundaries, sometimes substantially, to meet that day's science objectives. Hence, the recommended improvement associated with EVA traverse and timeline planning was to scale the size of the EVA stations according to the confidence in meeting the science objectives based on the precursor data available. This level of confidence is directly related to the precursor data type (*e.g.,* spectrum), resolution, and perspective (*e.g.,* overhead versus oblique). The total area planned to be visited during a particular EVA could then be scaled based on overall EVA time available. This change might enable better estimates of the search area needed to meet a given science objective and the amount of time that should be allocated to the different EVA phases and tasks.

The BASALT-2 EVA timelines were held relatively consistent across the four study conditions to enable the fairest comparison. However, timelines were preferentially designed to accommodate the high-latency (15 min OWLT) study conditions to provide as much candidate sample information as possible to the MSC before the MSC needed to send presampling or sampling leaderboard recommendations to the crew. Note that each EVA was associated with multiple science objectives (Table 7 in Brady *et al.,* 2019) and that each station was typically paired with a single science objective. BASALT-1 EVAs incorporated two-station timelines (Beaton *et al.,* 2017). It was identified after the BASALT-1 field test that a third station should be added for BASALT-2 EVAs so that, under high-latency conditions, the MSC could receive candidate sample information from at least two stations (as opposed to one) before needing to send their recommendations to the crew. One way to reconcile the recommended improvements from both BASALT-1 (*i.e.,* increase the number of stations visited prior to the MSC needing to send recommendations to the crew) and BASALT-2 (scale station size and number according to the precursor data available and science objectives to be met) is to design EVAs so that the crew plans to explore a single large region whose size is dictated by the precursor data available and particular science objectives needing to be met.

For *EV and IV roles, responsibility, and distribution of personnel,* the acceptability question of interest was “For future Mars missions with similar science objectives, how acceptable would it be to have two EV crewmembers, with one focused on science and one focused on operations, and two IV crewmembers, with one focused on science and one focused on operations?” This ConOps component was rated *totally acceptable scientifically* and *acceptable operationally*. With sufficient training and the right capabilities in the field and in the IV workstation, it was sufficient to have two EV crewmembers and two IV crewmembers, with one EV and IV focused on science and the other EV and IV focused on operations. With additional or better capabilities in the IV workstation, it was projected that it might be acceptable to only have one IV crewmember. For example, an enhanced EVA timeline management tool that incorporates more automation (*e.g.,* auto-logging of EVA start and stop times, auto-statusing of data transmission from the field to the MSC) and provides better summary snapshots of all the information needed to manage the EVA would increase IV crewmember efficiency (see Marquez *et al.* [2019] for further description of the IV workstation capabilities available during BASALT-2).

For *MSC roles, responsibilities, and distribution of personnel,* the acceptability question of interest was “For future Mars missions with similar science objectives, how acceptable would it be to have the BASALT MSC roles, responsibilities, and distribution of personnel?” This aspect of the ConOps was rated *scientifically and operationally acceptable*. The desired improvements identified were to more clearly define the roles, responsibilities, and distributions of personnel and to acknowledge that these are strongly dependent on (1) the capabilities provided (*e.g.,* if the necessary capabilities are provided in a manner in which they can be efficiently and effectively used, personnel requirements may be less) and (2) the level of training (*e.g.,* if sufficient training is provided, responsibilities may be completed more efficiently and effectively).

For *flight rules governing operations and safety specifically with respect to the advisory role of the MSC,* the acceptability question of interest was “For future Mars Missions with similar science objectives, how acceptable would it be to have the MSC act in an advisory role, with IV1 having ultimate authority and responsibility for operational EVA decisions and tactics and EV2 having ultimate authority and responsibility for scientific EVA decisions and tactics?” This was rated *totally acceptable scientifically and operationally*. Under the relatively long communication latencies (up to 22 min OWLT) and bandwidth constraints associated with Earth-Mars interactions, it is reasonable to presume that Earth-based scientists and mission support operators should not have ultimate authority, responsibility, and management over EVA operations. This is in stark contrast to current ISS EVA operations (and former Apollo EVA operations), in which the Mission Control Center plays a central role overseeing and guiding each EVA (see Beaton *et al.* [2019] for further discussion on the differences between Earth-based support for ISS EVA operations and support for future planetary EVA operations). For some science objectives associated with future exploration missions, however, enabling intra-EVA interactions between Mars-based crew and Earth-based science experts will likely enhance scientific discovery and return. A reasonable balance was achieved during BASALT-2 by establishing EV and IV responsibilities upfront and encouraging input from the MSC as much as was practical.

#### 3.2.2. Acceptability of the communication protocol and capability assessment of voice and text communication

The communication protocol assessed during BASALT-2 specifies the number and type of communication channels, the mode of communication, whether the communication is real-time or delayed, and the senders and receivers of those communication products. Results from previous analog communication protocol questionnaires (Chappell *et al.,* 2013b, 2016) and the BASALT-1 field test refined the communication protocol used during BASALT-2. Therefore, we only evaluated two questions with respect to BASALT-2 communication protocols: (1) “For future Mars Missions with similar science objectives, how acceptable would it be to have two SG communication channels, with SG-1 dedicated to EV-IV conversations with the MSC able to listen and SG-2 dedicated to IV-MSC conversations with EV not able to listen?” and (2) “For future Mars Missions with similar science objectives, how acceptable would it be to have IV1 and IV2 communicate with the MSC for operational and science matters, respectively, rather than the MSC communicating directly with EV1 and EV2? Does this change with only 1 IV crewmember?” The scientific and operational acceptability associated with each of these questions is presented in Table 4.

**Table 4.** Acceptability of the Communication Protocol

EV: extravehicular, IV: intravehicular, MSC: mission support center, Ops: operations, Sci: science, SG: space-to-ground.

The *SG-1 and SG-2* communication protocol component was rated *totally acceptable operationally* and *totally acceptable scientifically* with no recommended improvements. The acceptability of *IV as an intermediary between EV and the MSC* component was rated for two conditions: first, assuming two IV crewmembers were available (*i.e.,* the BASALT baseline) and second, assuming only one IV crewmember was available (the alternative condition proposed to be potentially acceptable during the ConOps acceptability discussion [see Section 3.2.1]). Under the single IV crewmember condition, it was assumed this individual would have access to the same set of capabilities. Both conditions were deemed *totally acceptable operationally* and *totally acceptable scientifically,* although it was noted that having two IV crewmembers might be slightly more acceptable during busier aspects of an EVA so that overall workload could be shared between two individuals, thereby potentially lowering the risk of EV crew idle time.

A key element of the communication protocol is the capabilities provided for communication among the EVA crewmembers and between the EVA crew and the MSC during the EVAs. Table 5 presents the capability assessment ratings provided by the scientists and operations personnel for intra-EVA voice and text communication among EV and IV crewmembers and the MSC. These results describe how essential and enabling these capabilities are presumed to be for future Mars exploration EVA with similar science objectives.

**Table 5.** Capability Assessment of Intra-EVA Voice and Text Communication

CA: capability assessment, E: EV/IV crew rating, EV: extravehicular, IV: intravehicular, M: MSC crew rating, MSC: mission support center, Ops: operations, Sci: science.

Voice communication between EV and IV crewmembers was rated as *essential/enabling* by the EVA crew. The MSC rated the ability to hear the EV/IV conversations (across time-delay) as *essential/enabling*. Voice communication from IV to the MSC was rated as *moderately enhancing*. IV crew noted that the ability to rapidly transmit voice memos to the MSC could be more efficient than typing text messages, especially under high workload situations. The MSC was best able to absorb this information when they were prompted by the IV crewmember (*e.g.,* with a few-second verbal countdown) that an important voice memo was incoming. The MSC rated this capability as *marginally enhancing*.

Voice communication from the MSC to IV crewmembers was rated as *little or no enhancement* by the EVA crew due to the fact that these messages were received across latency. Because the MSC had no way of knowing exactly what the crew would be doing when these messages would ultimately be received, the EVA crew discouraged voice communication from the MSC, as such interactions were distracting and potentially detrimental to the flow of science and operations. The MSC rated this capability as *marginally enhancing;* they could envision rare instances in which an “all stop” might be useful, for example if a given target was suddenly no longer worth investigating or a new science priority arose that required immediate attention.

Text communication between IV crewmembers and the MSC was rated as *essential/enabling*. The IV crew much preferred receiving text messages from the MSC over voice memos, as text messages could be addressed when workload allowed and be referred to later as needed. Of note, valuable features of the Playbook Mission Log text messaging system include its ability to keep high-priority messages at the top of the page and to play a Quindar tone when new messages are received (Marquez *et al.,* 2019).

#### 3.2.3. Acceptability and capability assessment of the capabilities

The capabilities incorporated in BASALT-2 stemmed from the results and lessons learned from previous analog missions and from initial BASALT planning that culminated in project functional requirements definition and capability implementation and integration. The ConOps and capabilities evaluated during BASALT focus on those that enable continuous, intra-EVA interactions between EVA crew and remote science experts separated by long communication latencies and limited bandwidth, which is the most stressing ConOps applicable to future human Mars missions.

Due to the extensive nature of the full list of capabilities incorporated during BASALT-2, not all capabilities were evaluated with the CA and acceptability rating scales. The capabilities prioritized for assessment were those that had been identified as potentially enhancing during previous analog missions, but had yet to be evaluated under Mars-relevant science objectives, as well as new capabilities identified and implemented during BASALT. These capabilities were organized into those that are relevant to pre-EVA planning and those that are relevant to intra-EVA operations; further subcategories are described in the following sections.

For each capability evaluated, simulation quality ratings were first collected to ensure that the manner in which the capability was defined and implemented was sufficient to provide meaningful CA and acceptability results. For BASALT-2, all capabilities evaluated had simulation quality ratings of 1, 2, or 3. CA ratings were then collected to project the level of mission enhancement for future Mars exploration EVA with similar science objectives under this particular ConOps. For capabilities that were rated at least marginally enhancing, operational and scientific acceptability ratings were collected and corresponding desired, warranted, and required improvements were documented.

##### 3.2.3.1. Pre-EVA: EVA planning capabilities

The CA and acceptability ratings for the capabilities relevant to pre-EVA planning are presented in Table 6. All CA ratings for the *EVA timeline* and *traverse planning* and *planned traverse path optimization* were provided by the MSC only, as the EVA crewmembers were not involved in pre-EVA planning.

**Table 6.** Capability Assessment and Acceptability of Pre-EVA EVA Planning Capabilities

CA: capability assessment, E: EV/IV crew rating, EVA: extravehicular activity, M: MSC rating, NR: not rated, Ops: operations, Sci: science.

The *EVA traverse planning* capability was rated *essential/enabling* by both the MSC operators and the scientists. *EVA timeline planning* was rated *essential/enabling* by the MSC operators and *significantly enhancing* by the MSC scientists. *EVA traverse planning* and *EVA timeline planning* were rated *acceptable* by both the MSC operators and scientists. The primary improvement desired for future Mars missions with similar science objectives was an increase in the resolution of precursor overhead imagery and multispectral data to 5–10 cm/pix. While this is a substantial increase in resolution compared to current Mars orbital assets (McEwen *et al.,* 2007; Murchie *et al.,* 2007), resolutions to this order of magnitude would enable scientists to more confidently pinpoint terrain features of interest for these types of science objectives, thereby improving timeline and traverse planning estimates and increasing crew efficiency in the field.

The *planned traverse path optimization* capability was rated *significantly enhancing* by both the MSC operators and the MSC scientists. Such a capability is important for automatically down-selecting the best potential traverse paths and enabling the MSC to estimate distances that the crew might need to cover and the duration that such traversing might take. However, the level of enhancement provided by such a capability is likely terrain dependent. For example, path optimization planning becomes more critical when the terrain does not allow direct visual inspection of the best routes in real-time, for example, because of large obstacles obstructing views or unseen changes in surface friability or surface temperature.

The features and functions evaluated for *planned traverse path optimization* were rated *unacceptable* by MSC operators and *borderline acceptable* by MSC scientists for future Mars exploration missions with similar science objectives. The primary improvement required for future path planning optimization algorithms is to incorporate multiple terrain features, including friability and surface temperature, in addition to terrain slope and projected EV crew metabolic cost. During BASALT-2, it was found that some locations of scientific interest where the terrain was relatively flat were in the end too fragile to support the weight of the crewmembers walking; this led to the occasional “punching through” and tripping and could have led to dangerous falls, risking injury to the crew and damage to hardware. Hence path planning optimization algorithms that incorporate multiple terrain features would improve traverse planning estimates and reduce overall risk in the field.

##### 3.2.3.2. Intra-EVA: EV support tool capabilities

The CA and acceptability ratings for capabilities relevant to supporting EV crewmembers in the field are presented in Table 7. The *navigation aids* provided EV crew with planned traverses, actual traverses, waypoints of interest, and mapped notes (*i.e.,* flags with brief descriptions at features of interest) added by the IV crew and MSC personnel throughout the EVA. These capabilities were rated *significantly enhancing* and *totally acceptable* with no improvements identified for future Mars exploration EVA.

**Table 7.** Capability Assessment and Acceptability of Intra-EVA EV Crew Support Tools

CA: capability assessment, E: EV and IV crew rating, M: MSC rating, RFID: radio frequency identifier, Ops: operations, Sci: science.

The *graphical display* projected the navigation aids listed above, text messages from the IV crew and the MSC, annotated images, and the field of view (FOV) of the EV chest-mounted cameras. This capability was rated *significantly enhancing* by the EVA crew and MSC scientists and *moderately enhancing* by the MSC operators. It was deemed more operationally efficient and scientifically effective for the EV crew to receive this information directly on a graphical display, versus receiving some of it via a text-only EVA cuff display (as was provided during DRATS 2010 [Hurtado *et al.,* 2013; Wright, 2016]) and the rest verbally from IV crewmembers (*e.g.,* descriptions of annotated images, whether targets of interest are being captured in the chest-mounted camera FOVs, navigation through ground-controlled approach instructions).

The *graphical display* was rated *operationally* and *scientifically acceptable* for future Mars EVA with several desired improvements noted. Improvements included the addition of easily accessible display pages with the current EVA's science objectives and summary task instructions, as well as critical EVA timeline countdown timers so that EV crew could see the remaining time for the current task and estimations for when they could expect to receive input from the MSC.

The *feature pointer* is a physical pointer (*e.g.,* a labeled meter stick), as opposed to an electronic pointer (*e.g.,* a laser pointer) that has been previously employed in other analogs (Abercromby *et al.,* 2013c). The purpose of incorporating a physical pointer was to provide both orientation and meter- and centimeter-scale size references in still imagery and video footage. The physical feature pointers employed during BASALT-2 were 1-m long, 1-in. diameter white PVC pipes with alternating 2-cm black and white stripes on one end. Two pointers oriented perpendicular to one another and aligned with cardinal directions were typically placed in the foreground of contextual photos. The pointers were also used to direct attention to specific features of interest; for close-up photos, one pointer was used as an orientation and scale bar, while a second was directed at the specific feature of interest. This capability was rated *essential/enabling* by all EV and IV crew and members of the MSC.

The *feature pointer* features and functions were rated *acceptable* scientifically and operationally with some desired improvements for future Mars missions. One notable improvement was to consider incorporating a more compact element, such as one that telescopes out to its full length. Alternatively, a feature pointer that unfolds into a t-shape or right angle would automatically encompass orthogonal orientations. Both improvements would enhance manageability for the user and accuracy.

The *feature marker* refers to a physical identification marker (as opposed to an electronic or virtual marker) that incorporates a unique label to unambiguously mark and identify a terrain feature of interest, as well as a scale bar, color bar, and orientation designator. This capability was rated *essential/enabling* by the EVA crew and the MSC for its ability to unambiguously designate targets of interest in the field. Such a capability with the associated features described above was rated scientifically and operationally *acceptable*. Desired improvements include ensuring an appropriate marker surface that minimizes glare and enables readability under all natural lighting and flash photography conditions, as well as the addition of a physical or virtual component, such as a vertical flag or electronic radio frequency identifier, so that the marker could be more easily relocated by the EV crew.

##### 3.2.3.3. Intra-EVA: Science instrument, video, and imagery capabilities

The capability assessment and acceptability ratings for intra-EVA video, still imagery, and scientific instrument data from the field are presented in Table 8. *Video from EV chest-mounted cameras* was rated *significantly enhancing* from an operations perspective (EV1, IV1, and MSC operators), *essential/enabling* from a science EVA crew perspective (EV2 and IV2), and *significantly enhancing* by MSC scientists. Operations personnel utilized the chest camera footage primarily for ongoing situational awareness of current EV tasks and EV whereabouts, while scientists used it to provide context for and detailed observation of terrain features of interest (*e.g.,* candidate sample locations).

**Table 8.** Capability Assessment and Acceptability of Intra-EVA Video, Still Imagery, and Scientific Instrument Data from the Field

CA: capability assessment, E: EV/IV crew rating, EV: extravehicular, FOV: field of view, IV: intravehicular, M: MSC rating, MSC: mission support center, Ops: operations, SA: situational awareness, Sci: science, UAS: unmanned aerial system.

The *video from EV chest-mounted cameras* capability was rated operationally and scientifically *acceptable*. Desired improvements included the ability to modify camera resolution, contrast, and dynamic range in real-time in the field to improve video quality under a wider range of environmental conditions (*e.g.,* light intensity, sun angle) and terrain variations. The ability to modify FOV so that a wider FOV could be employed during contextual surveys and narrower FOV could be used for close-up work was also desired. Other improvements include flexibility to vary the mounting location (*e.g.,* on the chest, helmet, or to be handheld) or to incorporate multiple cameras, which could be advantageous depending on the nature of the task being conducted. For example, a chest-level mount is useful when viewing the work area surrounding the EV crewmember's hands, which in a spacesuit will likely be limited to the area in front of the torso (Schmidt *et al.,* 2001). Helmet mounting could provide a view that is more aligned with what the crewmember is seeing, thereby potentially making it easier for IV crew and the MSC to correlate the incoming video footage with associated verbal descriptions. The ability to incorporate a handheld camera (or camera mounted on the back of the hand) might be particularly useful when close-up footage is desired of *in situ* rocks for which it is difficult to aim a chest- or helmet-mounted camera due to limitations in spacesuit range of motion (Schmidt *et al.,* 2001).

The *mobile SA video with position and orientation tracking* capability was rated *significantly enhancing* by all EVA crewmembers and MSC operators and scientists. The primary function of this capability was to provide continuous information to the IV crewmembers and the MSC regarding EV crewmembers' positions and orientations within the local terrain. For future planetary missions, it is possible that a similarly enhancing capability could be provided by a panoramic camera capturing “snapshots” (at some to-be-determined frequency and resolution) of the crew's activity (Abercromby *et al.,* 2013b); this alternative capability has yet to be tested and evaluated in a terrestrial analog that incorporates Mars-relevant science objectives, but is an important component of the BASALT MIP and future BASALT work.

The *mobile SA video with position and orientation tracking* capability was rated *borderline acceptable* with one warranted and several desired improvements identified. The warranted improvement noted was the inclusion of the mobile SA camera during EVA approach phases to improve general scientific context. Desired improvements included the provision of an overhead view, which could be provided by a higher mast-mounted camera system or possibly an unmanned aerial system (UAS) (Balaram *et al.,* 2018), and the potential for autotracking of the EV crew by the SA camera itself to reduce IV crew workload. Notably, the *mobile SA video with position and orientation tracking* capability implemented during BASALT-2 did not include IV-operable control of the camera's position and FOV or a camera with pan/tilt/zoom capabilities. Furthermore, it was operated from a height of only 1–2 m, as opposed to 3–4 m anticipated for rover mast-mounted systems (Hurtado *et al.*, 2013). It was confirmed during the consensus rating discussions that these baseline features and functions would indeed be valuable.

Under low-bandwidth test conditions, video footage from both the EV crew chest-mounted camera and the mobile SA camera was not sent to the MSC. In principle, operating under low-bandwidth conditions, compared with operating under high-bandwidth conditions, is a deficiency with no direct improvements. However, the EV and IV crew could partially make up for this by being strategic and judicious in their verbal communications, text messages, and still imagery. Furthermore, the crew could send low-resolution panoramic still images (*i.e.,* “snapshots”) from the chest-mounted and/or mobile SA cameras incrementally (*e.g.,* every few seconds or minutes) as bandwidth traffic allowed. Doing so would provide some level of real-time updates regarding the crew in their workplace for both real-time situational awareness and for archival purposes to document the EVA.

*High-resolution still imagery* collected during BASALT-2 EVAs was defined by an imagery protocol drafted by BASALT scientists, which consisted of systematic sets of contextual and close-up images of the surrounding terrain for each phase of the EVA plus any additional imagery deemed significant by the EV crew upon arrival at a location of interest (Stevens *et al.,* 2019). This capability was rated *essential/enabling* by EVA crewmembers and MSC scientists for meeting the Mars analog science objectives. The capability was rated *moderately enhancing* by MSC operators, who were primarily focused on operational matters regarding the crew, such as their health and well-being and overall EVA timeline progress. MSC operators wanted to maintain general situational awareness throughout the EVA, which was found to be accomplished more effectively by listening to the EV/IV conversations, sending the occasional text message, and by viewing the chest-mounted and SA camera video feeds (when bandwidth allowed) than by interpreting incoming still imagery.

The *high-resolution still imagery* was rated *acceptable operationally* and *borderline acceptable scientifically*. One warranted improvement identified was the inclusion of an automated indication to both EV and IV crewmembers that each image was successfully delivered. Another improvement noted was the ability to have manual control over exposure, depth of field, focal distance, and optical zoom to better account for variable lighting conditions, terrain features of interest, and the purpose of the image (*e.g.,* contextual versus close-up).

The *handheld scientific instrument data* capability comprises the features and functions of the handheld scientific instruments outlined in Table 1 and described in detail in the work of Sehlke *et al.* (2019). This capability was rated *essential/enabling* by MSC scientists and EVA crewmembers and *moderately enhancing* by MSC operators for future Mars EVA assuming similar science objectives and levels of precursor data. The level of mission enhancement for this capability is dependent on the particular science objectives of interest, as well as the level of precursor data available (including precursor data type, resolution, and viewing angle); different science objectives or levels of precursor data may render the need for handheld instruments in the field more or less essential and enabling. For BASALT-2 science objectives and precursor data available, the handheld instrument data was critical for informing sampling decisions (Sehlke *et al.,* 2019) and hence significantly impacted tactical, intra-EVA operations.

This capability was rated *borderline acceptable operationally* and *unacceptable scientifically* for future Mars exploration EVA. One warranted improvement was for the instrument to inform the EV crew of the proper sequence of scans during a given data collection session requiring multiple scans (*e.g.,* by different instruments) on the same surface; as each scan is conducted, the instrument should provide an indication of scan quality and inform the EV crew if a scan needs to be retaken. This would improve efficiency and accuracy in the field.

For Mars EVA with similar science objectives operating under this ConOps, the handheld scientific instrument data is critical to intra-EVA decision-making. Hence, required improvements to directly transmit the raw instrument data to the IV crew (who would likely have more sophisticated data-analysis and interpretation software in the IV workstation and could relay relevant aspects, including the raw data, to the MSC) and to provide an indication to the EV crew that the data was successfully delivered were identified. One potential alternative to these recommendations is for the instrument to analyze and interpret the data on board and to provide follow-on information and instructions directly to the EV crew.

##### 3.2.3.4. Intra-EVA: IV/MSC support capabilities

The capability assessment and acceptability ratings for intra-EVA IV and MSC support tools are presented in Table 9.

**Table 9.** Capability Assessment and Acceptability of Intra-EVA IV and MSC Support Tools

CA: capability assessment, E: EV/IV crew rating, EV: extravehicular, EVA: extravehicular activity, IV: intravehicular, LiDAR: light detection and ranging, M: MSC rating, Ops: operations, Sci: science.

*Geospatially linked electronic field notes* are electronic field notes, captured manually by IV crewmembers and the MSC in the Minerva software tool, that are visible to all and linked geospatially to EV crew position at the time the note is recorded (Marquez *et al.,* 2019). These field notes include scientific details regarding candidate samples and targets of opportunity. Electronic field notes are also used to track locations of interest such as traverse waypoints and positions of the mobile SA camera. Additional details regarding what these field notes entail and how they are recorded, organized, archived, and able to be searched are described in the works of Deans *et al.* (2017) and Marquez *et al.* (2019).

This capability was rated *essential/enabling* to the ConOps by all EVA crewmembers and the MSC. It was also rated *scientifically* and *operationally acceptable* with some desired improvements. One such improvement was the ability to incorporate a hands-free means of establishing these field notes to increase efficiency and reduce workload. For instance, adding a feature such as speech recognition (*e.g.,* “Create EV1 waypoint A1,” which would automatically create a waypoint called A1 at the current location of the EV1 crewmember) would enable EV or IV crew to quickly mark field locations of interest without requiring EV to take their hands off the tools they are currently using in the field or IV to alter their present IV workstation task. Another desired improvement was some means of maintaining a running tabulation of all notes related to a particular candidate sample that is automatically organized and readily visible to both the IV crew and the MSC. For the ConOps under evaluation, in which SG interactions are encouraged to enhance the science being conducted in the field, increasing efficiency and decreasing workload for IV and MSC personnel is critical for providing the best recommendations in the shortest amount of time when operating under communication latency and bandwidth constraints.

The *dynamic leaderboard* capability is a means of quickly, systematically, and dynamically ranking candidate targets of interest for presampling and sampling (Chappell *et al.,* 2016; Miller *et al.,* 2016; Stevens *et al.,* 2019). The concept has evolved through the BASALT project to include priority rankings, measures of strength of each ranking (*e.g.,* Priority 1 is much more highly desired than Priority 2, Priority 2 is only slightly more desirable than Priority 3), rationales associated with each ranking, and photos or video clips of each candidate. The leaderboard is traditionally compiled by MSC scientists who have a larger set of knowledge and capabilities at their disposal (*e.g.,* expertise to quickly read instrument spectral plots and additional software to interpret the incoming data). IV crewmembers often keep a crew-based leaderboard to track their own priorities, based on their ability to observe first-hand; IV crewmembers then cross-reference and update their crew leaderboard with MSC-provided recommendations as soon as they become available.

The *dynamic leaderboard* capability was rated *essential/enabling* by EV2 and IV2 science crewmembers and *significantly enhancing* by EV1 and IV1 operator crewmembers and members of the MSC for its ability to prioritize science and improve operational efficiency. The capability was rated *acceptable operationally* and *borderline acceptable scientifically* for future Mars missions. The most warranted improvement noted was to establish a centralized dynamic leaderboard (for instance, in a Minerva-like tool) that can be edited by the IV crew and the MSC and viewed by the EV crew. This could further improve efficiency in communication among all parties and in field operations. For example, a feature that allows the EV crew to view the current state of the leaderboard on a text or graphical display would alleviate the need for the IV crew to relay this information when they may be busy attending to other tasks. Adding an automated time stamp denoting when changes were made and by whom would eliminate confusion over whether a particular piece of information was known at the time of a given update or not. A final desired improvement was to link the leaderboard candidates to both the current EVA's science objectives and to the larger mission science objectives to help track progress toward meeting daily as well as overall mission science goals.

The *spatial and temporal synchronization of data* capability incorporates spatial and temporal synchronization of EV crew and mobile SA camera GPS positions, still imagery, scientific instrument data, and field notes. For BASALT-2, this was accomplished through the Minerva software tool (see Deans *et al.* [2017] and Marquez *et al.* [2019] for additional details regarding how the data synchronization was coordinated and how the associated data products were organized and archived). This capability was rated *essential/enabling* for science and *significantly enhancing* for operations for future Mars exploration EVA. It was also rated *borderline acceptable operationally* and *scientifically* with several warranted improvements identified. Noted improvements include automatic tagging of all data products according to the current EVA timeline phase to increase the efficiency in using the data sets both intra- and post-EVA, and the ability to “play back” portions of an EVA (defined by a selected time period or portion of the traverse) through accessing any data products (*e.g.,* video, still imagery, instrument data). These features could increase the efficiency of assimilating the incoming data by the MSC, which is particularly important to the ConOps under consideration.

The *image annotation* capability enables the IV crew and MSC members to overlay illustrations and footnotes onto still imagery to highlight features of interest. Common annotations include circling, crossing out, and arrows to direct attention to specific areas of the terrain. This capability was rated *moderately enhancing* for both science and operations, noting that this capability has the potential to moderately enhance scientific return and improve operational efficiency by reducing confusion on specific targets of interest. *Image annotation* was rated *totally acceptable scientifically* and *borderline acceptable operationally*. The primary warranted improvement identified for future Mars EVA was to ensure that the interface for creating and transmitting the image annotations could be conducted efficiently in a minimal number of steps.

The *tactical EVA timeline management* capability is a set of timeline management features used by the IV crew and the MSC to track and project EVA timeline progress against a predefined baseline. Key features and functions of this capability include displaying the sequence of planned EVA phases and planned phase durations, recording actual phase durations, projecting future phase start times based on actual phase durations, displaying running phase elapsed time (PET, the time since the start of the EVA) clocks and countdown timers for key EVA deadlines (*e.g.,* time until the MSC should send sampling recommendations to minimize the chances of the EV crew incurring idle time), and providing space for taking notes associated with each EVA phase (see Table 1). The tactical EVA timeline management capability was rated *essential/enabling* by EV1 and IV1 crewmembers and the MSC and *significantly enhancing* by EV2 and IV2 crewmembers.

The features associated with this capability were rated *acceptable* with desired improvements by both science and operations crew for future Mars exploration EVA. To reduce overall workload and the number of independent systems that need to be monitored and interfaced with during the EVA, this capability would ideally be integrated into a Minerva-like program (as opposed to being run in a separate spreadsheet tool). Additionally, it would be advantageous for the EV crew to be able to view some of these features directly, including the PET running clocks and EV-specific countdown timers on their graphical wrist display, thereby reducing the need for IV to relay all critical aspects of timeline progress.

The *+/− ∼1 m position tracking* capability included the ability to track the position of EV crewmembers, the mobile SA camera, and other terrain features of interest to within approximately 1 m of their actual positions in the field. For future Mars exploration EVA, this capability was rated *essential/enabling* for science and operations and *acceptable operationally* and *scientifically*. The primary improvement identified was to ensure a means for reducing random positioning system uncertainty in the logging and display of mobile entities (*e.g.,* incorporating sufficient filtering algorithms to reduce apparent motion of EV crew when they were actually standing still). Note that although BASALT used GPS to track positions, the results from this study do not necessarily recommend a Mars-based GPS for human exploration; other means of relative position tracking on the surface could be employed, such as inertial measurements and visual odometry (Powell *et al.,* 2010), which may be more cost effective, accurate, and deemed similarly mission enhancing and acceptable.

The *EVA traverse* and *timeline replanning* capabilities include rapid replanning of EVA traverses and timelines in real-time during the EVA. *EVA traverse replanning* was rated *essential/enabling* by EV2 and IV2 crewmembers and MSC scientists and *moderately enhancing* by EV1 and IV1 crew members and MSC operators. The *EVA timeline replanning* capability was rated *essential/enabling* by EV2 and IV2 crewmembers, *significantly enhancing* by MSC scientists, and *moderately enhancing* by EV1 and IV1 crew members and MSC operators. *EVA traverse replanning* and *EVA timeline replanning* were both rated *acceptable operationally* and *scientifically*. Improvements identified for future Mars exploration EVA with similar science objectives include enhanced precursor data, as described in Section 3.2.3.1 (higher-resolution overhead imagery [5–10 cm/pix] and the inclusion of multispectral data [0.4–3.9 μm at 5–10 cm/pix]), as well as real-time integration of additional data products gathered in the field upon arrival of the crew, such as oblique imagery, 360° panoramas, and LiDAR data from 3–4 m above ground level and with minimum resolution of 1 cm at a distance of 10 m. These additional capabilities are described further in Section 3.3.

### 3.3. Capability assessment of additional Mars-forward capabilities

As discussed in Section 3.2, some of the originally planned baseline capabilities could not be implemented during BASALT-2 and hence were not evaluated from an acceptability standpoint. Each of these baseline capabilities, however, had been deemed at least moderately enhancing during previous analog missions, so we collected capability assessment ratings to determine their level of potential mission enhancement for future Mars exploration EVA under this ConOps with similar science objectives. The CA ratings for these capabilities are provided in Table 10 and designated with a ^x^.

**Table 10.** Capability Assessment of Mars-Forward Capabilities Evaluated but not Implemented During BASALT-2

^x^ : capability identified before BASALT-2 was conducted, ^*^: new capability identified during BASALT-2, NR^a^: capability not rated because it did not apply operations or science. NR^b^: capability not rated because evaluator did not have sufficient experience with the capability to provide a judicious rating.

3D: three dimensional, AR: augmented reality, CA: capability assessment, E: EV/IV crew rating, EV: extravehicular, EVA: extravehicular activity, IV: intravehicular, LiDAR: light detection and ranging, M: MSC rating, MR: mixed reality, NR: not rated, Ops: operations, Sci: science, SG: space-to-ground, VR: virtual reality, xGDS: exploration ground data system.

Some of the improvements identified during the BASALT-2 acceptability ratings process came in the form of recommendations for new capabilities, as described previously in Section 3.2. At the end of the BASALT-2 field test, we compiled a list of these new capabilities and evaluated their anticipated level of enhancement for future Mars EVA through the CA rating process. These results are also presented in Table 10 and are designated with a *.

Note that some of the capabilities listed in Table 10 were given “no rating” by the BASALT-2 operators and/or scientists. This was either because the evaluators felt that this capability did not influence their particular area of expertise (*i.e.,* not applicable to either the operations or the science, designated with an ^a^) or the evaluators felt that they did not have sufficient experience with the capability to provide a judicious rating (designated with a ^b^). Section 4.3 further discusses a few of these capabilities in relation to forward work.

## 4. Discussion

### 4.1. Science Operations research questions

The BASALT project is investigating five strategic Science Operations research questions, presented in Section 1.2, regarding ConOps and capabilities for future Mars exploration EVA while conducting Mars-relevant terrestrial fieldwork. Each successive BASALT field test has iteratively worked toward addressing these questions. Science Operations results from the first BASALT field test are presented in the work of Beaton *et al.* (2017). The following sections summarize the Science Operations results of BASALT-2, acknowledge several study limitations, and propose additional forward work.

#### 4.1.1. Baseline ConOps, systems, and communication protocol acceptability

The BASALT Science Operations research question 1A asks *Do the baseline ConOps, systems, and communication protocols developed and tested during previous NASA analogs work acceptably during real scientific exploration? What improvements are desired, warranted, or required?* The acceptability of the baseline ConOps, systems, and communication protocols developed and tested during previous analogs (Beaton *et al.,* 2019) was rated as described in Sections 3.2.1, 3.2.3, and 3.2.2, respectively. Each of these sections also describes improvements identified during the rating process that might make the ConOps, system, or communication protocol more acceptable and notes inherent deficiencies that were identified.

The portion of question 1A regarding the *acceptability of the baseline ConOps* is addressed via the acceptability of the individual components identified in Table 3. Three of the four baseline ConOps components (*EV/IV roles, responsibilities, and distribution of personnel; MSC roles, responsibilities, and distribution of personnel;* and *flight rules governing operations and safety, specifically with respect to the advisory role of the MSC*) were rated acceptable (acceptability ratings ≤4). The ConOps component *EVA tasks and their organization into EVA timelines and traverses* was rated borderline acceptable due to (1) the inherent deficiencies noted for EVAs operating under high communication latencies (under which improvements could not be recommended since communication latency cannot be modified) and (2) the recommendation to scale the size and number of EVA stations based on the level of precursor data available and the science objectives needing to be met. Other improvements could have been proposed, such as collecting higher-resolution precursor data to match the science objectives. The key for supporting a ConOps that focuses on enabling intra-EVA SG interactions is to design EVA timelines and traverse plans so that Earth-based science experts can receive critical data products from the field across latency and have sufficient time to assimilate that incoming data to inform recommendations (which is highly dependent on the number of personnel and capabilities available) and send those recommendations back to the crew across latency, all of which should be conducted without the crew incurring idle time.

The *acceptability of the systems* portion of question 1A is addressed in the acceptability ratings of the capabilities implemented for BASALT-2. The features and functions column associated with the 24 capabilities listed in Table 1 serves as the baseline set of high-level, Mars-forward functional and performance requirements recommendations for science operations systems development. *Desired, warranted,* and *required* improvements were identified for capabilities rated less than *totally acceptable* (Tables 6–9). These improvements serve as modified or new requirements that are intended to make the ConOps more acceptable.

The *acceptability of the communication protocol* portion of question 1A is addressed through the acceptability ratings shown in Table 4. The communication protocol was rated *totally acceptable* with no identified improvements or deficiencies.

Science operations research question 1B asks *Do the baseline ConOps, systems, and communication protocols remain acceptable across the range of Mars mission communication latencies and bandwidth considerations? What improvements are desired, warranted, or required?* Overall, the numerical ratings of acceptability of the ConOps, systems, and communication protocols did not vary with latency or bandwidth, although more work is needed to truly address the effects of varying bandwidth on acceptability. Inherent deficiencies were identified in high versus low latency conditions and high versus low bandwidth conditions, as described in Section 3.2. While deficiencies typically lead to decreased acceptability, the deficiencies related to latency and bandwidth offset one another such that numerical ratings remained the same.

For instance, given the fact that the same baseline EVA timelines were employed for both the low and high communication latency study conditions and that the timelines were designed such that the MSC could assimilate data from two stations prior to sending recommendations to the crew under the high latency EVAs, one could argue that EVAs associated with a lower latency could have been designed more efficiently; this argues for better acceptability associated with high latency EVAs. However, low latency EVAs consistently enable more GAT, which argues that EVAs associated with lower latencies may be more acceptable for providing tactical, intra-EVA phase input.

Furthermore, because a number of baseline capabilities were not able to be implemented and a means for dynamic bandwidth management was not in place, the MSC did not have a complete set of data products coming from the field that needed to be prioritized and likely down-selected under the low-bandwidth EVAs. The general consensus was that in order to properly test and evaluate the effects of bandwidth on science return, the MSC needed access to the full set of capabilities plus some means of manual or automatic bandwidth management.

Overall, the findings for BASALT research questions 1A and 1B validate many of the acceptability results from previous analog tests that investigated similar ConOps, systems, and communication protocols, but did not incorporate Mars-relevant field science (Chappell *et al.,* 2016; Miller *et al.,* 2016). Our results also demonstrate that continuous and meaningful input from remote science experts is achievable during exploration EVA, even under long communication latencies and bandwidth limitations. EVA timelines can be designed to facilitate these SG interactions without incurring EV and IV crewmember idle time, but the overall acceptability is strongly contingent on the capabilities available to the crew and the MSC (Beaton *et al.,* 2019). The following section addresses which capabilities are essential to the success of this ConOps.

#### 4.1.2. Level of mission enhancement of baseline capabilities

Science Operations research question 2A asks *Which capabilities (utilized by “Mars” EV and IV crewmembers and “Earth” MSC personnel) are enabling and enhancing for scientific exploration EVA?* All capabilities incorporated and evaluated during BASALT-2 were rated at least marginally enhancing scientifically and/or operationally for future Mars exploration EVA (Section 3.2.3 and Tables 6–9). Many of these capabilities were rated *essential/enabling* (*i.e.,* it is impossible or highly inadvisable to conduct a mission without these capabilities) or *significantly enhancing* (*i.e.,* likely to significantly enhance one or more aspects of the mission): precursor imagery for traverse and timeline planning, planned traverse path optimization, intra-EVA voice communication between EV and IV, text communication between IV and the MSC, high-resolution still imagery from EV crew handheld cameras, video footage from EV chest-mounted cameras and a mobile situational awareness camera, scientific instrument data, EV crew navigation aids, EV crew graphical display, EVA feature pointer, EVA feature marker, geospatially linked electronic field notes, dynamic leaderboard, spatial and temporal synchronization of data, tactical EVA timeline management, +/− ∼1 m position tracking, and EVA traverse and timeline replanning. By rating these capabilities enabling and enhancing, we can propose candidate requirements through the combination of their tested features and functions (listed in Table 1) and the improvements identified for each capability (listed in Tables 6–9).

Research question 2B asks *Does the degree of enabling and enhancing (for these capabilities) vary as communication latency and bandwidth availability change?* No changes in levels of mission enhancement afforded by the capabilities were found across the latency and bandwidth conditions evaluated during this field test. Additional recommendations for addressing this question are provided in Section 4.3.

### 4.2. Study limitations

Beaton *et al.* (2019) describe several study limitations for BASALT-2 Science Operations research including (1) inconsistent EVA execution by the three different EV/IV/MSC teams, (2) hardware/software troubleshooting and communication network instabilities resulting in intermittent availability of some capabilities during the EVAs (which led to overall simulation quality ratings of 3), and (3) the lack of the complete baseline architecture intended for the MIP. Missing elements from the MIP included a mast-mounted camera system capable of collecting high-resolution panoramic imagery and LiDAR data in real-time during the EVA (Beaton *et al.,* 2019). This limitation may have affected the ability to evaluate high- versus low-bandwidth conditions since the large file sizes associated with these capabilities would have exceeded the low-bandwidth threshold for data passing from space to ground. Had these capabilities been available, the MSC would have needed to make additional strategic decisions regarding which data products to prioritize under low-bandwidth conditions during each phase of the EVA. This, in turn, may have affected the consensus-rated degree of enhancement of some capabilities under high- versus low-bandwidth test conditions, and is hence a subject of forward work (Section 4.3).

### 4.3. Forward work

In addition to providing design and implementation recommendations for Mars EVA ConOps and capabilities, the work conducted during BASALT-2 informed the inclusion, implementation, and testing of capabilities for the BASALT-3 field test. BASALT-3, which took place in November 2017, sought to (1) address the *desired, warranted,* and *required* improvements proposed during BASALT-2, (2) incorporate missing and several new capabilities identified during BASALT-2, and (3) reevaluate the acceptability of the ConOps and capabilities and the level of mission enhancement of the capabilities in light of these changes. The complete set of BASALT-3 results will be published in subsequent papers, but notable implementation improvements and evaluations for BASALT-3 are outlined in the following paragraphs.

BASALT-3 EVA traverses incorporated the exploration of a single larger station, whose baseline size was dictated by the confidence in meeting that EVA's science objectives based on the level of precursor data available. Corresponding EVA timelines were designed so that the MSC had sufficient GAT to provide meaningful input to the crew without the crew incurring idle time. All BASALT-3 EVAs were conducted under a single communication latency (5 min) and without SG bandwidth restrictions.

Since architectural and technological assumptions regarding bandwidth availability for future human Mars missions are still largely to be determined (Seibert *et al.,* 2019), BASALT-3 focused on simply evaluating the level of mission enhancement and acceptability of capabilities previously deemed useful (*i.e.,* during BASALT-2 and during previous analog missions that did not evaluate these capabilities during actual, Mars-relevant scientific exploration) for science and science operations. The premise was that if these capabilities were found to be non-mission-enhancing or unacceptable under unconstrained bandwidth conditions, they would presumably be even less mission-enhancing and further unacceptable under restricted bandwidth conditions. Capabilities found to be potentially mission-enhancing could then be reevaluated during future field tests once Mars-Earth communication architecture and infrastructure are better defined. File sizes and data rates for each capability evaluated during BASALT-3 were recorded and analyzed to determine their bandwidth impact on potential communication architectures and to inform recommendations for additional functional and performance requirements.

BASALT-3 EVA study conditions focused on different sets of capabilities available to the EVA crewmembers and to the MSC during precursor EVA planning and during the EVAs. Initial EVAs evaluated the baseline set of capabilities only, which included high-resolution 360° panoramic still imagery and mobile automated LiDAR. Later EVAs incorporated new capabilities identified, but not tested, during BASALT-2. Table 10 identifies five potentially *essential/enabling* capabilities and eighteen potentially *significantly enhancing* capabilities. Several of the new capabilities incorporated and evaluated during BASALT-3 included virtual training environments and head-mounted display technologies for the EV crew, mixed reality three-dimensional terrain models, and telepresence systems for the IV crew and the MSC to “join” the EV crew in the field.

## 5. Conclusions

The BASALT project seeks to inform Mars EVA science operations through the iterative testing and evaluation of exploration ConOps and capabilities. The BASALT-2 field test design and execution built upon the results and lessons learned of BASALT-1, as well as previous NASA analog missions. The ConOps, systems, and communication protocols assessed during BASALT-2 were found to be *borderline acceptable* for future Mars exploration EVAs that incorporate intra-EVA interactions with an Earth-based science team. Multiple corresponding desired, warranted, and required improvements were proposed, many of which were incorporated and reevaluated during the BASALT-3 field test.

BASALT-2 also assessed the level of mission enhancement of 41 Mars-forward capabilities, identifying which capabilities were most likely to be essential and enabling for future Mars EVA and which capabilities may no longer be worth pursuing. Those capabilities that were found to be the most significantly enhancing formed the basis for BASALT-3 testing and will remain the focus of future work.

In summary, BASALT Science Operations objectives seek to build critical knowledge related to the design and requirement-generation challenges associated with future science-driven planetary EVA. Our efforts focus on informing exploration EVA ConOps and capabilities, as well as future terrestrial testing to further vet and refine details. The results from the BASALT-2 and BASALT-3 field tests are currently being integrated into EVA ConOps documents, owned and maintained by the NASA JSC EVA office.

## Abbreviations Used

BASALT: Biologic Analog Science Associated with Lava Terrains
CA: capability assessment
ConOps: concepts of operations
DEM: digital elevation model
DRATS: Desert Research and Technology Studies
EAMD: Exploration Analog and Mission Development
EV: extravehicular
EVA: extravehicular activity
EVIB: extravehicular informatics backpack
FOV: field of view
GAT: ground assimilation time
in-sim: in-simulation
IV: intravehicular
JSC: Johnson Space Center
LiDAR: light detection and ranging
MIP: Mobile Instrument Platform
MSC: Mission Support Center
NEEMO: NASA Extreme Environment Mission Operations
OWLT: one-way light time
PET: phase elapsed time
PLRP: Pavilion Lake Research Project
SA: situational awareness
SCICOM: science communicator
SG: space-to-ground
UAS: unmanned aerial system
xGDS: Exploration Ground Data System
x-sim: out-of-simulation

## References

[B1] Abercromby A., Cupples S., Rajulu S., Buffington J., Norcross J., and Chappell S. (2015) Integrated extravehicular activity (EVA) human research plan: 2016. In *46^th^ International Conference on Environmental Systems*, Vienna, Austria

[B2] Abercromby A.F., Gernhardt M.L., and Litaker H.L. (2010) Desert Research and Technology Studies (DRATS) 2008: Evaluation of Small Pressurized Rover and Unpressurized Rover Prototype Vehicles in a Lunar Analog Environment, NASA Technical Report TP-2010-216136, NASA Johnson Space Center, Houston, TX

[B3] Abercromby A.F.J., Gernhardt M.L., and Litaker H. (2012) Desert Research and Technology Studies (DRATS) 2009: A 14-Day Evaluation of the Space Exploration Vehicle Prototype in a Lunar Analog Environment, NASA Technical Report TP-2012-217360, NASA Johnson Space Center, Houston, TX

[B4] Abercromby A.F., Chappell S.P., and Gernhardt M.L. (2013a) Desert RATS 2011: human and robotic exploration of near-Earth asteroids. Acta Astronautica 91:34–48

[B5] Abercromby A.F., Gernhardt M.L., and Jadwick J. (2013b) Evaluation of dual multi-mission space exploration vehicle operations during simulated planetary surface exploration. Acta Astronautica 90:203–214

[B6] Abercromby A.F.J., Chappell S.P., Litaker H.L., Reagan M.L., and Gernhardt M. (2013c) NASA Research and Technology Studies (RATS) 2012: virtual simulation and evaluation of human and robotic systems for exploration of near-Earth asteroids. In *43^rd^ International Conference on Environmental Systems*, Vail, CO, doi:10.2514/6.2013-3506

[B7] Balaram B., Canham T., Duncan C., Grip H.F., Johnson W., Maki J., Quon A., Stern R., and Zhu D. (2018) Mars helicopter technology demonstrator. In *Atmospheric Flight Mechanics Conference*, AIAA, Kissimmee, FL, doi:10.2514/6.2018-0023

[B8] Beaton K.H., Chappell S.P., Abercromby A.F., Miller M.J., Kobs Nawotniak S., Hughes S.S., Brady A., and Lim D.S. (2017) Extravehicular activity operations concepts under communication latency and bandwidth constraints. In *2017 IEEE Aerospace Conference*, IEEE, Big Sky, MT, doi:10.1109/AERO.2017.7943570

[B9] Beaton K.H., Chappell S.P., Abercromby A.F.J., Miller M.J., Kobs Nawotniak S.E., Brady A.L., Stevens A.H., Payler S.J., Hughes S.S., and Lim D.S.S. (2019) Using science-driven analog research to investigate extravehicular activity science operations concepts and capabilities for human planetary exploration. Astrobiology 19:300–320, doi:10.1089/ast.2018.1861 30840499 PMC6442238

[B10] Beaty D., Niles P., Hays L., Bass D., Bell M.S., Bleacher J., Cabrol N.A., Conrad P., Eppler D., Hamilton V., Head J., Kare M., Levy J., Lyons T., Rafkin S., Rice J., and Rice M. (2015) Candidate Scientific Objectives for the Human Exploration of Mars, and the Implications for the Identification of Martian Exploration Zones, MEPAG, Jet Propulsion Laboratory, Pasadena, CA

[B11] Brady A.L., Kobs Nawotniak S.E., Hughes S.S., Payler S.J., Stevens A.H., Cockell C.S., Elphic R.C., Sehlke A., Haberle C.W., Slater G.F., and Lim D.S.S. (2019) Strategic planning insights for future science-driven extravehicular activity on Mars. Astrobiology 19:347–368, doi:10.1089/ast.2018.1850 30840500 PMC6442241

[B12] Chappell S.P., Abercromby A.F., Todd W.L., and Gernhardt M.L. (2011) NEEMO 14: Evaluation of Human Performance for Rover, Cargo Lander, Crew Lander, and Exploration Tasks in Simulated Partial Gravity, NASA Technical Report TP-2011-216152, NASA Johnson Space Center, Houston, TX

[B13] Chappell S.P., Abercromby A.F., and Gernhardt M.L. (2013a) NEEMO 15: evaluation of human exploration systems for near-Earth asteroids. Acta Astronautica 89:166–178

[B14] Chappell S.P., Abercromby A.F.J., Reagan M., and Gernhardt M. (2013b) NEEMO 16: evaluation of systems for human exploration of near-Earth asteroids. In *43^rd^ International Conference on Environmental Systems*, AIAA, Vail, CO, doi:10.2514/6.2013-3508

[B15] Chappell S.P., Graff T.G., Beaton K.H., Abercromby A.J.F., Halcon C., Miller M.J., and Gernhardt M.L. (2016) NEEMO 18–20: analog testing for mitigation of communication latency during human space exploration. In *2016 IEEE Aerospace Conference*, IEEE, Big Sky, MT, doi:10.1109/AERO.2016.7500717

[B16] Crusan J.C., Craig D.A., and Herrmann N.B. (2017) NASA's deep space habitation strategy. In *2017 IEEE Aerospace Conference*, IEEE, Big Sky, MT, doi:10.1109/AERO.2017.7943624

[B17] Deans M., Marquez J.J., Cohen T., Miller M.J., Deliz I., Hillenius S., Hoffman J., Lee Y.J., Lees D., and Norheim J. (2017) Minerva: user-centered science operations software capability for future human exploration. In *2017 IEEE Aerospace Conference*, IEEE, Big Sky, MT, doi:10.1109/AERO.2017.7943609

[B18] Frank J., McGuire K., R. Moses H., and Stephenson J. (2016) Developing decision aids to enable human spaceflight autonomy. AI Magazine 4, doi:10.1609/aimag.v37i4.2683

[B19] Gernhardt M.L., Bekdash O.S., Litaker H.L., Chappell S.P., Beaton K.H., Newton C., Crues E.Z., and Abercromby A.F.J. (2017) Mars ascent vehicle sizing, habitability, and commonality in NASA's evolvable Mars campaign. In *2017 IEEE Aerospace Conference*, IEEE, Big Sky, MT, doi:10.1109/AERO.2017.7943726

[B20] Hodges K. and Schmitt H. (2011) A new paradigm for advanced planetary field geology developed through analog experiments on Earth. Geological Society of America Special Papers 483:17–31

[B21] Hughes S.S., Haberle C.W., Kobs Nawotniak S.E., Sehlke A., Garry W.B., Elphic R.C., Payler S.J., Stevens A.H., Cockell C.S., Brady A.L., Heldmann J.L., and Lim D.S.S. (2019) Basaltic terrains in Idaho and Hawai‘i as planetary analogs for Mars geology and astrobiology. Astrobiology 19:260–283, doi:10.1089/ast.2018.1847 30339033 PMC6442300

[B22] Hurtado J.M., Young K., Bleacher J.E., Garry W.B., and Rice J.W. (2013) Field geologic observation and sample collection strategies for planetary surface exploration: insights from the 2010 Desert RATS geologist crewmembers. Acta Astronautica 90:344–355

[B23] ISECG. (2018) Global Exploration Roadmap, International Space Exploration Coordination Group, Mulheim/Ruhr, Germany

[B24] Jaumann R., Neukum G., Behnke T., Duxbury T.C., Eichentopf K., Flohrer J., Gasselt S., Giese B., Gwinner K., and Hauber E. (2007) The high-resolution stereo camera (HRSC) experiment on Mars Express: instrument aspects and experiment conduct from interplanetary cruise through the nominal mission. Planet Space Sci 55:928–952

[B25] Likert R. (1932) A technique for the measurement of attitudes. Archives of Psychology 22:1–55

[B26] Lim D.S.S., Abercromby A.F.J., Kobs Nawotniak S.E., Lees D.S., Miller M.J., Brady A.L., Miller M.J., Mirmalek Z., Sehlke A., Payler S.J., Stevens A.H., Haberle C.W., Beaton K.H., Chappell S.P., Hughes S.S., Cockell C.S., Elphic R.C., Downs M.T., Heldmann J.L., and the BASALT Team (2019) The BASALT research program: designing and developing mission elements in support of human scientific exploration of Mars. Astrobiology 19:245–259, doi:10.1089/ast.2018.1869 30840510 PMC6442272

[B27] Litaker H., Chen M., Howard R., and Cloyd B. (2012) Human Factors Assessment for the Space Exploration Vehicle (SEV) GEN 2A Habitable Volume Three Day Study—Research and Technology Studies (RATS) Phase 2, NASA, Washington, DC

[B28] Litaker H.L., Abercromby A.F.J., Moore N.R., Chappell S.P., Howard R.L., and Klein J.S. (2013) Assessment of the Habitable Airlock (HAL) and the Alternate Multiple Mission Space Exploration Vehicle (AMMSEV) Configurations in the NASA Neutral Buoyancy Laboratory (NBL), NASA Johnson Space Center, Houston, TX

[B29] Litaker H.L. Jr., Thompson S.G., Szabo R., Twyford E.S., Conlee C.S., and Howard R.L. Jr. (2013) Dual rover human habitation field study. Acta Astronautica 90:378–390

[B30] Marquez J.J., Pyrzak G., Hashemi S., Ahmed S., McMillin K.E., Medwid J.D., Chen D., and Hurtle E. (2013) Supporting real-time operations and execution through timeline and scheduling aids. In *Proceedings of the 43^rd^ International Conference on Environmental Systems*, American Institute of Aeronautics and Astronautics, Reston, VA, doi:10.2514/6.2013-3519

[B31] Marquez J.J., Miller M.J., Cohen T., Deliz I., Lees D.S., Zheng J., Lee Y.J., Kanefsky B., Norheim J., Deans M., and Hillenius S. (2019) Future needs for science-driven geospatial and temporal extravehicular activity planning and execution. Astrobiology 19:440–461, doi:10.1089/ast.2018.1838 30840505 PMC6442304

[B32] McEwen A.S., Eliason E.M., Bergstrom J.W., Bridges N.T., Hansen C.J., Delamere W.A., Grant J.A., Gulick V.C., Herkenhoff K.E., Keszthelyi L., Kirk R.L., Mellon M.T., Squyres S.W., Thomas N., and Weitz C.M. (2007) Mars Reconnaissance Orbiter's High Resolution Imaging Science Experiment (HiRISE). J Geophys Res: Planets 112, doi:10.1029/2005JE002605

[B33] Miller M.J., Lim D.S., Brady A.L., Cardman Z., Bell E., Garry W.B., Reid D., Chappell S., and Abercromby A.F. (2016) PLRP-3: Operational perspectives conducting science-driven extravehicular activity with communications latency. In *2015 IEEE Aerospace Conference*, IEEE, Big Sky, MT, doi:10.1109/AERO.2016.7500643

[B34] Miller M.J., Miller M.J., Santiago-Materese D., Seibert M.A., and Lim D.S.S. (2019) A flexible telecommunication architecture for human planetary exploration based on the BASALT science-driven Mars analog. Astrobiology 19:478–496, doi:10.1089/ast.2018.1906 30840502

[B35] Murchie S., Arvidson R., Bedini P., Beisser K., Bibring J.P., Bishop J., Boldt J., Cavender P., Choo T., and Clancy R. (2007) Compact reconnaissance imaging spectrometer for Mars (CRISM) on Mars Reconnaissance Orbiter (MRO). J Geophys Res: Planets 112, doi:10.1029/2006JE002682

[B36] NAC. (2018) Human Exploration and Operations Committee Report, NASA Advisory Council Meeting, NASA, Washington, DC

[B37] NASA. (2015) NASA Technology Roadmaps, TA5: Communications, Navigation, and Orbital Debris Tracking and Characterization Systems, NASA, Washington, DC

[B38] Niles P., Beaty D., Hays L., Bass D., Bell M.S., Bleacher J., Cabrol N.A., Conrad P., Eppler D., and Hamilton V. (2017) Scientific investigations associated with the human exploration of Mars in the next 35 years [abstract 8167]. In *Planetary Science Vision 2050 Workshop*, Lunar and Planetary Institute, Houston, TX

[B39] Pohlkamp K.M., Mauldin J., and Frank J.D. (2015) Demonstrating autonomous mission operations onboard the International Space Station. In *SPACE 2015 Conference and Exposition*, AIAA, Pasadena, CA, doi:10.2514/6.2015-4646

[B40] Powell M., Crockett T., Shams K., and Norris J. (2010) Geologic mapping in Mars rover operations. In *SpaceOps 2010 Conference*, AIAA, Huntsville, AL, doi:10.2514/6.2010-1998

[B41] Rush J., Israel D., Ramos C., Deutsch L., Dennehy N., and Seibert M. (2012) Communication and Navigation Systems Roadmap, Technology Area 5, NASA, Washington, DC

[B42] Schmidt P., Newman D., and Hodgson E. (2001) Modeling space suit mobility: applications to design and operations [2001-01-2162]. In *31^st^ International Conference on Environmental Systems*, SAE, Orlando, FL

[B43] Sehlke A., Mirmalek Z., Burtt D., Haberle C.W., Santiago-Materese D., Kobs Nawotniak S.E., Hughes S.S., Garry W.B., Bramall N., Brown A.J., Heldmann J.L., and Lim D.S.S. (2019) Requirements for portable instrument suites during human scientific exploration of Mars. Astrobiology 19:401–425, doi:10.1089/ast.2018.1841 30840506 PMC6442242

[B44] Seibert M.A., Lim D.S.S., Miller M.J., Santiago-Materese D., and Downs M.T. (2019) Developing future deep-space telecommunications architectures: a historical look at the benefits of analog research on the development of Solar System internetworking for future human spaceflight. Astrobiology 19:462–477, doi:10.1089/ast.2018.1915 30840504 PMC6442236

[B45] Stevens A.H., Kobs Nawotniak S.E., Garry W.B., Payler S.J., Brady A.L., Miller M.J., Beaton K.H., Cockell C.S., and Lim D.S.S. (2019) Tactical scientific decision-making during crewed astrobiology Mars missions. Astrobiology 19:369–386, doi:10.1089/ast.2018.1837 30840503 PMC6442282

[B46] Wright T.W. (2016) Advanced Spacesuit Informatics Software Design for Power, Avionics and Software Version 2.0, NASA/TM - 2016-219149, NASA Glenn Research Center, Cleveland, OH

